# Targeting SERT promotes tryptophan metabolism: mechanisms and implications in colon cancer treatment

**DOI:** 10.1186/s13046-021-01971-1

**Published:** 2021-05-18

**Authors:** Di Ye, Huanji Xu, Hongwei Xia, Chenliang Zhang, Qiulin Tang, Feng Bi

**Affiliations:** Department of Medical Oncology, Cancer Center and Laboratory of Molecular, Targeted Therapy in Oncology, West China Hospital, Sichuan University, Chengdu, 610041 Sichuan Province China

**Keywords:** Serotonin, SERT, Tryptophan, mTORC1, Trametinib

## Abstract

**Background:**

Serotonin signaling has been associated with tumorigenesis and tumor progression. Targeting the serotonin transporter to block serotonin cellular uptake confers antineoplastic effects in various tumors, including colon cancer. However, the antineoplastic mechanism of serotonin transporter inhibition and serotonin metabolism alterations in the absence of serotonin transporter have not been elucidated, especially in colon cancer, which might limit anti-tumor effects associating with targeting serotonin transporter.

**Methods:**

The promotion in the uptake and catabolism of extracellular tryptophan and targeting serotonin transporter was detected by using quantitative reverse-transcription polymerase chain reaction, western blotting and liquid chromatography tandem mass spectrometry. Western blotting Immunoprecipitation and immunofluorescence was utilized to research the serotonylation of mTOR by serotonin and serotonin transporter inhibition. The primary mouse model, homograft model and tissue microarry was used to explore the tryptophan pathway in colon cancer. The cell viability assay, western blotting, xenograft and primary colon cancer mouse model were used to identify whether the combination of sertraline and tryptophan restriction had a synergistic effect.

**Results:**

Targeting serotonin transporter through genetic ablation or pharmacological inhibition in vitro and in vivo induced a compensatory effect by promoting the uptake and catabolism of extracellular tryptophan in colon cancer. Mechanistically, targeting serotonin transporter suppressed mTOR serotonylation, leading to mTOR inactivation and increased tryptophan uptake. In turn, this process promoted serotonin biosynthesis and oncogenic metabolite kynurenine production through enhanced tryptophan catabolism. Tryptophan deprivation, or blocking its uptake by using trametinib, a MEK inhibitor, can sensitize colon cancer to selective serotonin reuptake inhibitors.

**Conclusions:**

The present study elucidated a novel feedback mechanism involved in the regulation of serotonin homeostasis and suggested innovative strategies for selective serotonin reuptake inhibitors-based treatment of colon cancer.

**Supplementary Information:**

The online version contains supplementary material available at 10.1186/s13046-021-01971-1.

## Background

Serotonin, also referred to as 5-hydroxytryptamine (5-HT), is a neurotransmitter and vasoconstrictor that is synthesized in the central and peripheral systems. The intestine is the main source of serotonin in the periphery [1]. It is an important enteric mucosal signaling molecule that has been implicated in the pathophysiology of various gut disorders, including colon cancer [2–4]. Studies have reported that serotonin is a key signaling molecule in the tumorigenesis, growth and metastasis of colon cancer [3, 5–8]. Serotonin reuptake transporters (SERT) and membrane serotonin receptors (HTR) are involved in the regulation of tumor development.

Uptake of serotonin by SERT is an indispensable process in serotonin recycling and degradation. Studies have also reported that intracellular serotonin is an important signal regulator that forms covalent bonds (also known as serotonylation) with various proteins, such as RhoA, Rab, and histones [9–11]. This process, which is catalyzed by transglutaminase 2 (TG2) through transamidation, regulates various protein characteristics and gene transcription. Studies have reported that selective serotonin reuptake inhibitors (SSRIs), such as sertraline, exert antineoplastic effects by inhibiting SERT activity.

Two main pathways regulate intracellular serotonin availability for tumor cells; SERT-mediated exogenous uptake and endogenous synthesis. Serotonin biosynthesis proceeds through Tryptophan hydroxylase (TPH)-catalyzed Tryptophan (Trp) catabolism [12]. Trp is an essential amino acid (EAA) that is imported into cells by membrane amino acid transports solute carrier 1A5 (SLC1A5; also named ASCT2) and solute carrier 7A5 (SLC7A5; also named LAT1) [13–15]. In addition to being involved in protein synthesis, Trp can also undergo TPH or indoleamine 2,3-dioxygenase (IDO)-catalyzed catabolism, thereby producing serotonin and the oncogenic metabolite kynurenine (Kyn) in tumor cells [16–18].

A limited number of studies have evaluated the mechanisms through which cells regulate the homeostasis of serotonin metabolism and corresponding alterations in the absence of SERT, especially in colon cancer. In this study, we found that targeting SERT induced a compensatory effect by promoting Trp uptake and catabolism in colon cancer. Mechanistically, targeting SERT suppressed mTOR serotonylation, leading to its inactivation. Inactivation of mTOR upregulated SLC1A5 expression, which elevated Trp uptake and catabolism. In turn, this process promoted serotonin biosynthesis and oncogenic kynurenine production, thereby maintaining colon cancer survival and vitality. Moreover, Trp deprivation or blocking its uptake using trametinib, a MEK inhibitor, sensitized colon cancer to SERT inhibition. In conclusion, this study elucidates on the mechanisms involved in the regulation of serotonin homeostasis and suggests innovative strategies for SSRIs-based treatment of colon cancer.

## Materials and methods

### Cell culture and reagents

Human colon cancer cell lines (SW480 and HCT116) were cultured in Dulbecco’s modified Eagle medium (DMEM, Gibco, USA) supplemented with 10% fetal bovine serum (FBS, Gibco, USA), 100 mU/mL of penicillin, and 100 μg/mL streptomycin. Incubation was done at 37 °C in a 5% CO_2_ atmosphere. Trametinib (GSK1120212) was purchased from Selleckchem, USA; sertraline was purchased from MedChemExpress, USA; serotonin, tryptophan and dansylcadaverine were purchased from Sigma, USA; asenapine was purchased from Topscience; antibodies against SLC1A5, mTOR, P70S6K, and TG2 were purchased from Proteintech, USA; antibodies against p-P70S6K (Thr389), PARP1, and Bcl-xL were purchased from CST, USA while anti-GAPDH was purchased from ZSGB-BIO, China.

### Cell transfection

SiRNAs against SERT, TG2 and the negative control were designed and synthesized by RIBOBIO, China. Transfection was performed using a Lipofectamine® RNAiMAX reagent (Invitrogen, USA) according to the manufacturer’s protocol. Sequences of the siRNAs used were:

Negative control: 5′-UUCUCCGAACGUGUCACGUTT-3′;

siSERT: 5′-TTCACAGTGCTCGGTTACA-3′;

siTG2: 5′-GCAACCTTCTCATCGAGTA-3′;

### Immunoprecipitation assays

Cells were lysed using a specific lysis buffer for IP (Beyotime). Whole-cell lysates were incubated overnight at 4 °C in the presence of antibodies against control IgG or mTOR. Then, the antibody-protein complex was incubated in the presence of protein A/G beads (Selleck). The antibody-protein-beads complex were washed three times, and analyzed by western blotting.

### Immunofluorescence staining

Cells were attached with 4% paraformaldehyde for 15 min and then permeability with 0.2% Triton X-100 for 3 min. After blocking with 1% bovine serum albumin (BSA) for 30 min, cells were incubated overnight at 4 °C in the presence of primary antibodies. Then, they were incubated in the presence of Alexa Fluor 488- or 594-conjugated secondary antibodies for 1.5 h at room temperature. Cells were washed using PBS and stained with DAPI (5 μg/ml) for 5 min (Invitrogen). Immunofluorescence was observed using a fluorescence microscope (Eclipse 80i, Nikon, Japan) at 200 and 400 magnifications.

### RNA extraction, cDNA synthesis and quantitative real-time PCR

Total RNA from cells and tissues were extracted using a total RNA Isolation Kit (Firegene, China), and reverse transcribed using the PrimeScript RT-PCR kit (Takara). Real-time PCR was performed using SYBR Premix Ex Taq (Takara) on a 7500 Real-time PCR system (Applied Biosystems) following the recommended thermal cycling conditions. Relative mRNA expression levels were calculated using the 2 (−ΔΔCT) method and normalized to GAPDH mRNA levels. Gene-specific primers were purchased from Sangon, and the sequences are shown in supplementary (Table 1).

### Cell proliferation and colony formation assays

Cells were plated in 96-well plates at a density of 4000-6000 cells per well and cultured overnight. Then, transfection was performed for 48 h after which the cell proliferation rate was evaluated using the Cell Counting Kit-8 (Dojingdo, Kumamoto, Japan). Regarding the colony formation assay, cells were seeded in 6-well plates in a regular medium and incubated with drugs for 5-7 days. Then, they were fixed in paraformaldehyde (4%), dyed with 0.5% crystal violet, and imaged using a digital camera.

### Colon cancer tissue microarray and immunohistochemistry

The human colon cancer tissue microarrays were prepared by Shanghai Outdo Biotech, China. Participants involved in this study provided an informed consent, and this study was ethically approved by the Ethics Committee of Taizhou Hospital of Zhejiang Province. The IHC scores were calculated based on the percentage of positively-stained cells and staining intensity, as previously described [19]. A 0-4 total score was regarded as low expression while a 5-12 score was regarded as high expression.

### LC-MS/MS quantification of Trp metabolites

For cell line studies, cells were plated in 10 cm wells at a density of 1 × 10^6^ cells/well and cultured in DMEM supplemented with 10% serum for 24 h. Then, the medium was replaced by DMEM supplemented with 15 uM sertraline. After another 12 h, cells were collected and extracted for metabolite measurements. Metabolites were normalized to total cell number. For animal serum analysis, blood was obtained from mice orbits in accordance with animal ethics requirements. After erythrocyte agglutination, serum was extracted and stored at − 80 °C before quantification.

### Animal models

Animals were bred and housed under pathogen-free conditions. All animal experiments were performed according to the guidelines of Sichuan University’s Institutional Animal Care and Use Committee (Cheng Du, China). SERT-KO mice were obtained from the Jackson Lab (Bar Harbor, ME, USA). SERT wildtype (SERT-WT) mice were utilized as controls. For the primary colon tumorigenesis model, a single intraperitoneal (i.p.) injection of azoxymethane (AOM, 10 mg/kg; Sigma-Aldrich, Heidenheim, BW, DE) and three cycles of dextran sodium sulfate (DSS, 1.5% Sigma-Aldrich) in drinking water were applied as previously described [20]. For allograft tumors, female BALB/c nude mice (4–5 weeks) were purchased from Beijing HFK Bioscience. Approximately 1 × 10^6^ HCT116 cells were subcutaneously injected into the right flank of mice. Once the tumors were palpable, mice were randomly distributed into six groups (*n* = 5), including the ethanol control group (DMSO), sertraline group (30 mg/kg), trametinib (2 mg/kg), PTR group, sertraline plus PTR group and the sertraline plus trametinib group. One week before injection, the diet of all mice was switched to PTR or to a specific control diet. Tumor volumes were serially measured after every 2 days.

### Statistical analysis

Comparisons between groups in cell proliferation assays and gene expression analysis were performed by GraphPad Prism 5 using the student’s t-test (two-tailed). Statistical significance was set at *p* < 0.05 (*), *p* < 0.01 (**) and *p* < 0.001 (***). Every experiment was performed in triplicate.

## Results

### SERT inhibition promoted Trp uptake and catabolism in vitro

**Fig. 1:**
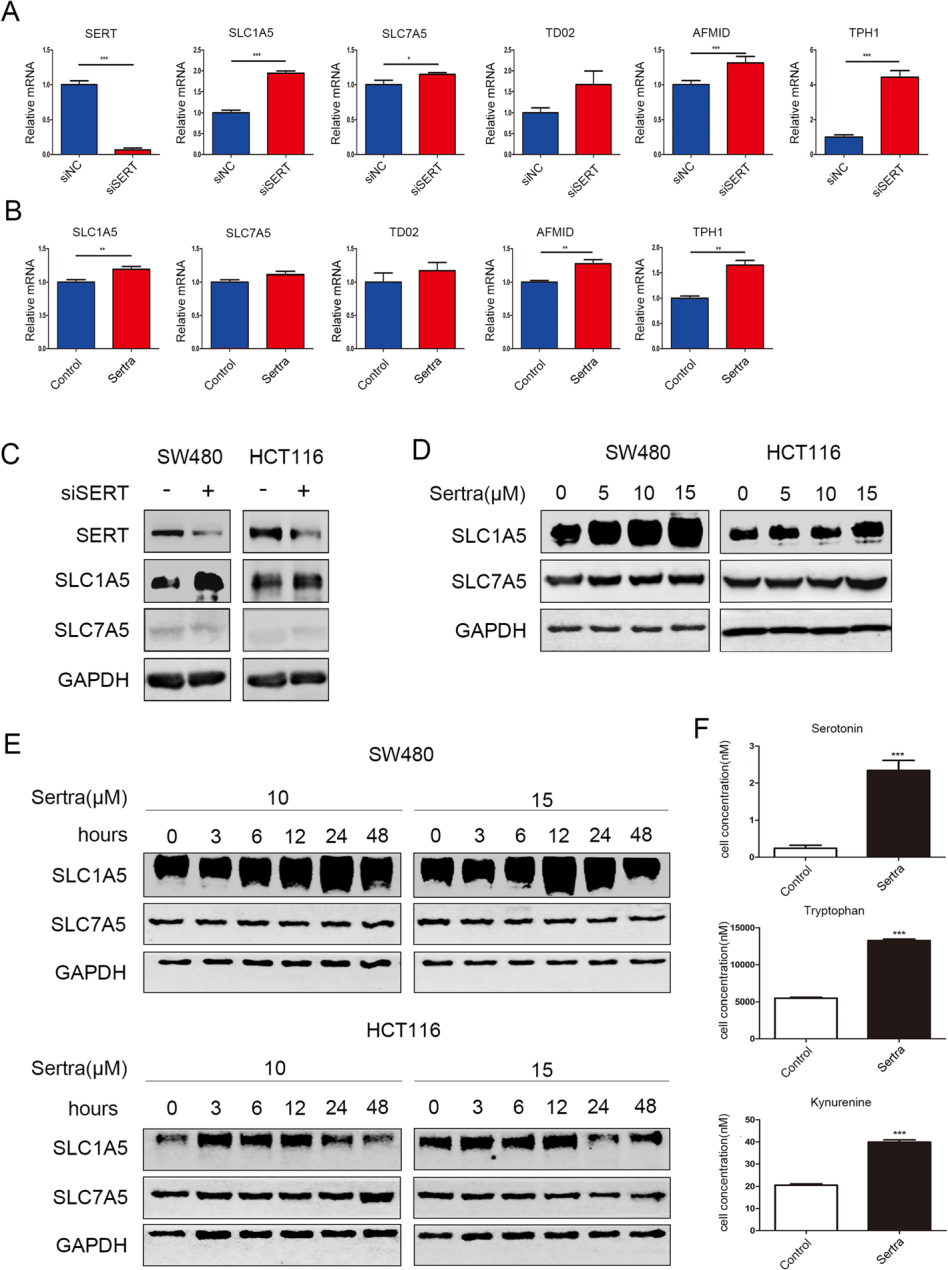
SERT inhibition promotes Trp uptake and catabolism in vitro*.* **a** An RT-PCR for SERT, SLC1A5, SLC7A5, TDO2, AFMID and TPHI mRNA expression in SW480 cells transfected with siSERT or negative control (48 h). The mRNA expressions were normalized to GAPDH. **b** An RT-PCR for SLC1A5, SLC7A5, TDO2, AFMID and TPHI mRNA expression in SW480 cells treated with sertraline (15 μM, 12 h) or DMSO. The mRNA expressions were normalized to GAPDH. **c**, **d** WB for SERT, SLC1A5, SLC7A5 in SW480 and HCT116 cells transfected with siSERT or negative control for 72 h, or treated with increasing concentrations of sertraline (5–15 μM) for 12 h. **e** WB for SLC1A5, SLC7A5 in SW480 and HCT116 cells treated with increasing concentrations (5–15 μM) of sertraline for increasing hours (0-48 h). **f** SW480 cells were harvested 12 h after sertraline treatment for metabolite extraction. Intracellular concentrations of Trp, Kyn, and serotonin were quantified by LCMS/MS. Cell numbers were determined and metabolite concentrations were normalized to cell counts from the same sample. *p < 0.05; ** *p* < 0.05; ***p < 0.001 using the Student’s t test (two-tailed)

To determine how colon cancer cells maintain serotonin metabolic homeostasis in the absence of SERT, serotonin biosynthesis from Trp catabolism was assessed. The mRNA expression levels of Trp transporters (SLC1A5 and SLC7A5) as well as those of Trp catabolism-related enzymes (TPH1, TDO2, and AFMID) were dysregulated after silencing SERT or after its inhibition by sertraline in HCT116 and SW480 cells (Fig. 1a, b, Additional file 1: Figure S1A, S1B). Western blot confirmed the elevated protein levels of SLC1A5 in SW480 and HCT116 cells (Fig. 1c, d), while SLC7A5 protein levels were not affected (Fig. 1e). In addition, intracellular concentrations of Trp and Kyn were elevated after sertraline treatment (Fig. 1f). Although sertraline inhibited serotonin uptake, intracellular serotonin concentration was elevated after sertraline treatment, which was attributed to increased Trp catabolism and subsequent serotonin biosynthesis (Fig. 1f). Kyn and serotonin have long been recognized as proliferation-promoting molecules, a conclusion that was verified in our in vitro experiment (Additional file 2: Figure S2A, S2B). These findings imply that SERT inhibition promotes Trp uptake and catabolism, which may contribute to the maintenance of intracellular concentrations of serotonin.

### Trp catabolism is activated in SERT-deleted colon cancer in vivo

**Fig. 2:**
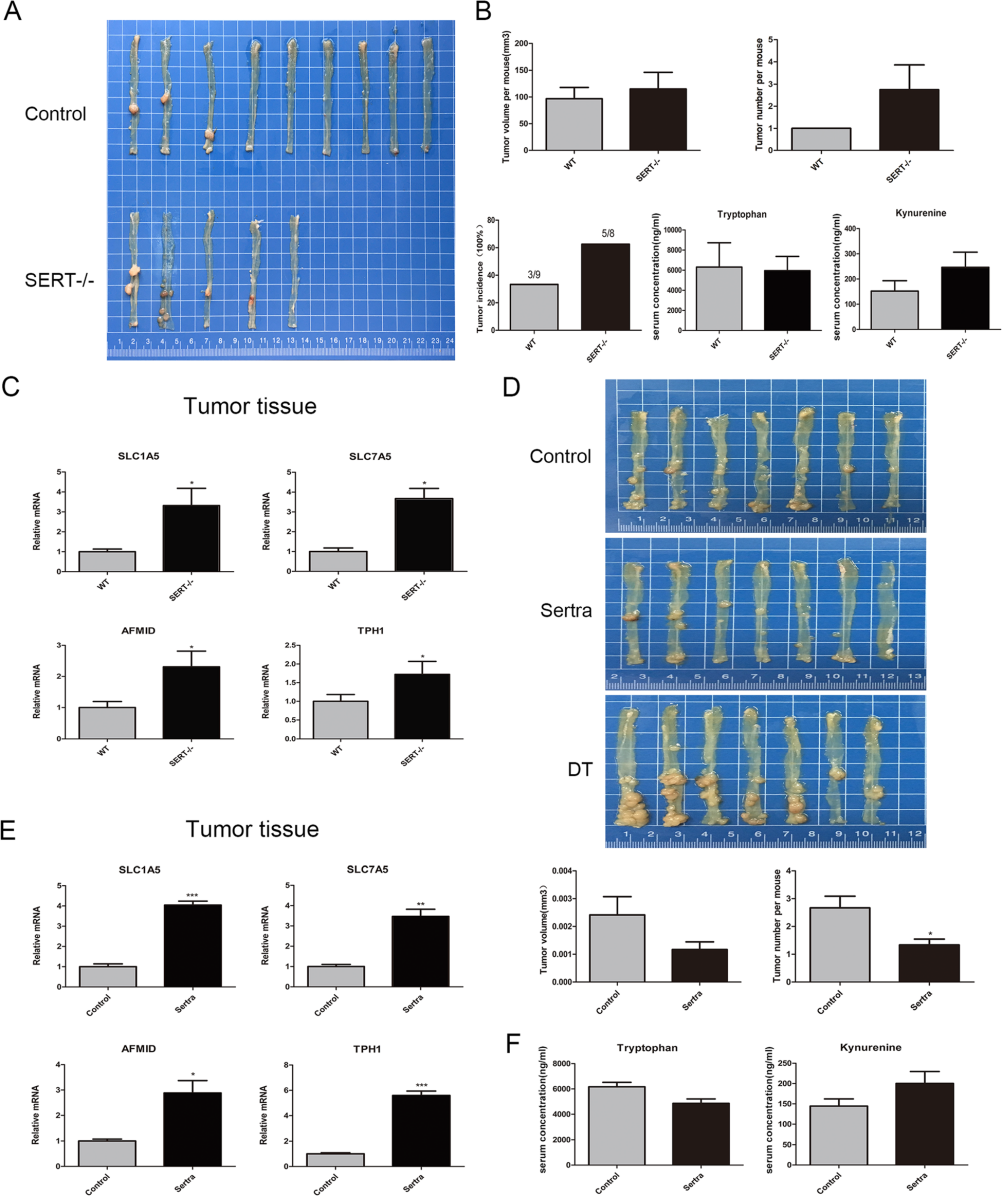
Trp catabolism was activated in SERT-deleted CC in vivo*.* **a** AOM-DSS method was used to induce primary colon cancer in SERT-KO and SERT-WT mice. Mice were injected with AOM (10 mg/kg) and then subjected to three cycles of DSS (1.5%) in drinking water. Fourteen weeks after AOM injection, tumors were dissected and their volumes and number determined. **b** A linear graph of tumor incidence, average number and tumor area for each group (mean ± SD). Serum levels of Trp, Kyn and serotonin in SERT-WT and SERT-KO mice in normal and AOM/DSS group were detected using LCMS/MS. **c** RT-PCR for mRNA expression of the indicated Trp transporters and enzymes in tumor tissues of SERT-WT and SERT-KO mice. **d** The primary colon cancer model was established by AOM-DSS method in SERT-WT mice. One month after AOM injection, mice were randomly distributed into three groups (*n* = 7) including the control group, sertraline group and double tryptophan (DT) group. The DT diet (4 mg/kg Trp) or a relevant control diet (2 mg/kg Trp) were provided during normal drinking water without DSS. The sertraline group and control group were subjected to sertraline (30 mg/kg) or saline treatment. After 14 weeks, tumors were dissected and their volumes and number determined. The bar graph shows the tumor volumes and number in each group (mean ± SD). **e** RT-PCR for expression of the indicated Trp transporters and enzymes in tumor tissues of control and sertraline group mice. **f** Serum levels of Trp, Kyn of control and sertraline group were detected using LCMS/MS. *p < 0.05; ** *p* < 0.05; *** *p*<0.001 using Student’s test (two-tailed)

To confirm the activation of Trp catabolism after SERT inhibition in vivo, AOM/DSS-induced colon cancer models in SERT-WT mice and SERT-KO mice were constructed. Surprisingly, SERT-KO mice developed more tumors than SERT-WT mice, suggesting that SERT deletion promotes colon cancer tumorigenesis, however, there was no difference in tumor volumes between the two groups (Fig. 2a, b). mRNA expression levels of SLC1A5, SLC7A5, AFMID, and TPH1 were all elevated in SERT-KO tumors compared to those in SERT-WT tumors (Fig. 2c). However, compared to those of SERT-WT normal colonic tissues, mRNA levels were not significantly elevated in SERT-KO normal intestinal tissues (Additional file 3: Figure S3A). Similar results were observed in AOM/DSS-induced colon cancer models in SERT-WT mice with sertraline treatment (Fig. 2e). In contrast to SERT gene deletion, sertraline significantly suppressed tumor volumes and numbers, probably due to its inhibition of other potential oncogenic targets (Fig. 2d). Serum levels of Kyn were elevated in SERT-KO mice and SERT-WT mice that had been treated with sertraline, however, Trp levels were suppressed, suggesting that SERT-inhibited CC might consume more Trp and produce more Kyn (Fig. 2b, f).

### SERT inhibition suppresses mTOR signaling to promote Trp uptake and catabolism

**Fig. 3:**
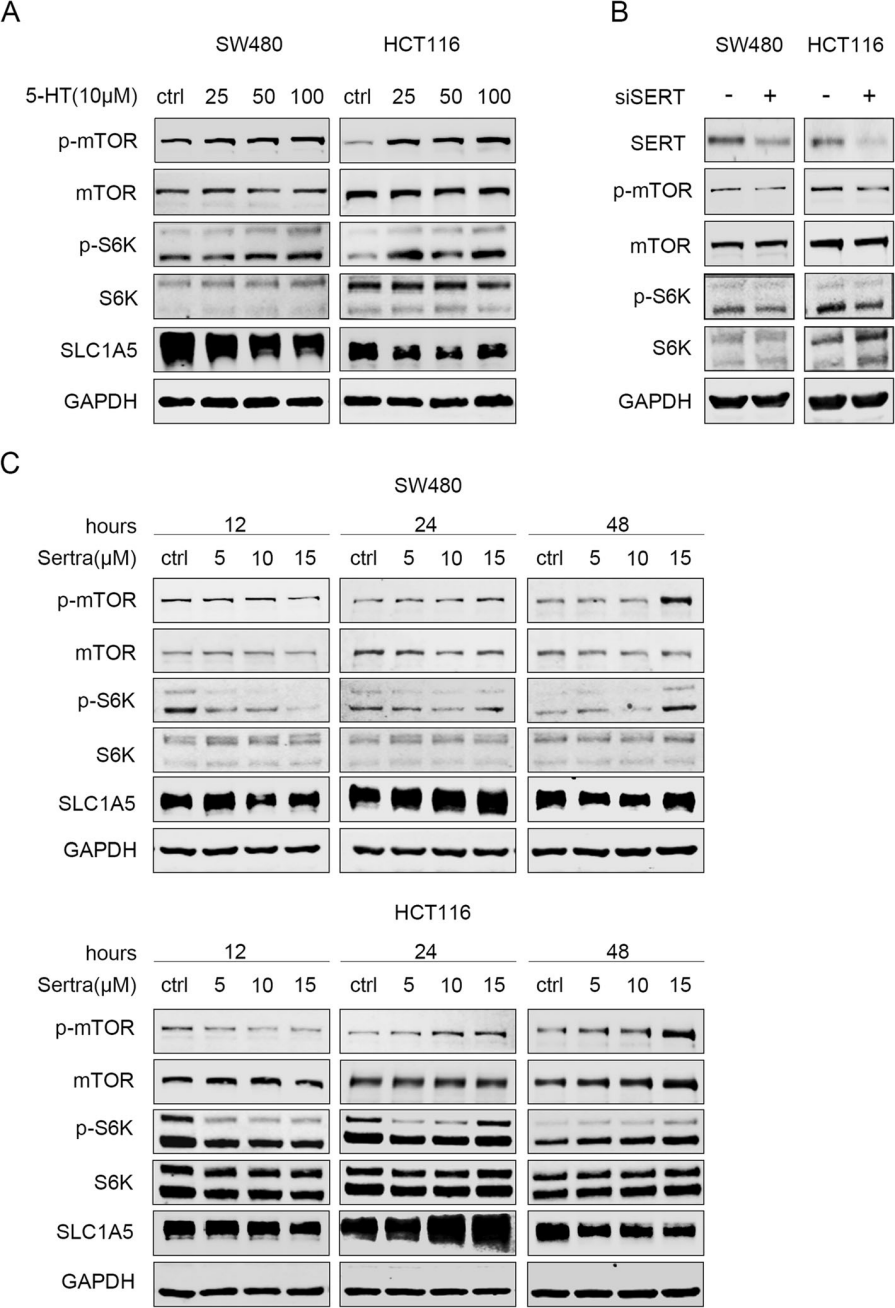
Inhibition of SERT blocks activation of the mTOR pathway. **a** WB for mTOR, S6K and SLC1A5 in HCT116 and SW480 cells treated with increasing concentrations of serotonin (25–100 μM) for 12 h. **b** WB for mTOR, S6K and SLC1A5 in HCT116 and SW480 cells transfected with siSERT or negative siNC for 48 h. **c** WB for mTOR, S6K and SLC1A5 in HCT116 and SW480 cells treated with increasing concentrations (5–15 μM) and process time (0–48 h) of sertraline

To investigate the underlying mechanisms that promote Trp uptake and catabolism, the supposed pathway was analyzed. It has been reported that serotonin can activate mTOR and elevate the expression of S6K, the major target of mTOR complex 1. Consistent with the findings of previous studies, our results showed that serotonin activates the mTOR pathway and elevates the expression levels of p-mTOR and p-S6K (Fig. 3a) while inhibition of SERT by siRNA or sertraline suppressed the expression of p-mTOR and p-S6K (Fig. 3b, c). Moreover, p-mTOR and p-S6K protein levels were suppressed at first, after which they were reactivated by sertraline treatment, while SLC1A5 protein levels were elevated at first, and then decreased (Fig. 3c). Inhibition of mTOR by rapamycin promoted SLC1A5 expression, while activation of mTOR by MHY1485 inhibited SLC1A5 expression (Additional file 4: Figure S4A, S4B). Besides SERT, dopamine transporters (DAT) and polyspecific organic cation transporters (OCT-1 and OCT-3) can also transport serotonin with lower affinities and selectivity than SERT. To determine whether SLC1A5 up-regulation was due to the blockade of serotonin reuptake, serotonin was supplemented under the conditions of SERT inhibition, which partially reversed SLC1A5 up-regulation (Additional file 4: Figure S4C, S4D). These findings indicate that SERT inhibition suppresses mTOR signaling to promote Trp uptake and catabolism.

### Serotonin activates mTORC1 through serotonylation

**Fig. 4:**
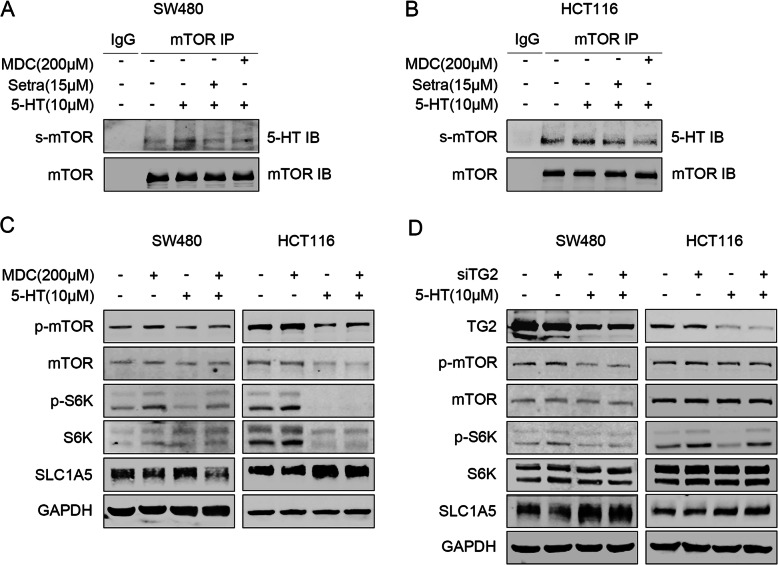
Serotonin activates mTORC1 through serotonylation. **a**, **b** SW480 and HCT116 cells were treated with serotonin for 10 min with or without MDC (TG2 inhibitor) (200 μM) or sertraline (15 μM) pretreated for 30 min. Anti-mTOR was used for IP. Western Blot were probed with anti-serotonin. **c** WB for mTOR, S6K and SLC1A5 in HCT116 and SW480 cells treated with or without serotonin in the presence or absence of MDC. **d** WB for mTOR, S6K and SLC1A5 in HCT116 and SW480 cells treated with or without serotonin after transfection with siSERT or negative control

Serotonin modifies some proteins through TG2-mediated serotonylation. To investigate the mechanisms through which serotonin-induces mTOR activation, mTOR serotonylation was evaluated. We confirmed the existence of serotonylation in mTOR. Importantly, serotonin treatment elevated the levels of serotonylated mTOR, and this process was reversed by the TG2 inhibitor, MDC, or sertraline treatment (Fig. 4a, b). In addition, TG2 inhibition suppressed the mTOR activity and reversed the activation of mTOR by serotonin stimulation (Fig. 4c, d). Previous studies reported that serotonin activates mTOR through a 5-HTR-dependent pathway. However, we found that mTOR activity was not altered after treatment with a broad-spectrum 5-HTRs inhibitor, Anazepin (Additional file 5: Figure S5). These findings indicate that SERT inhibition suppressed the levels of serotonylated mTOR, thereby inhibiting mTOR activity and promoting Trp uptake and catabolism.

### Endogenous and exogenous serotonin have different subcellular localizations

**Fig. 5:**
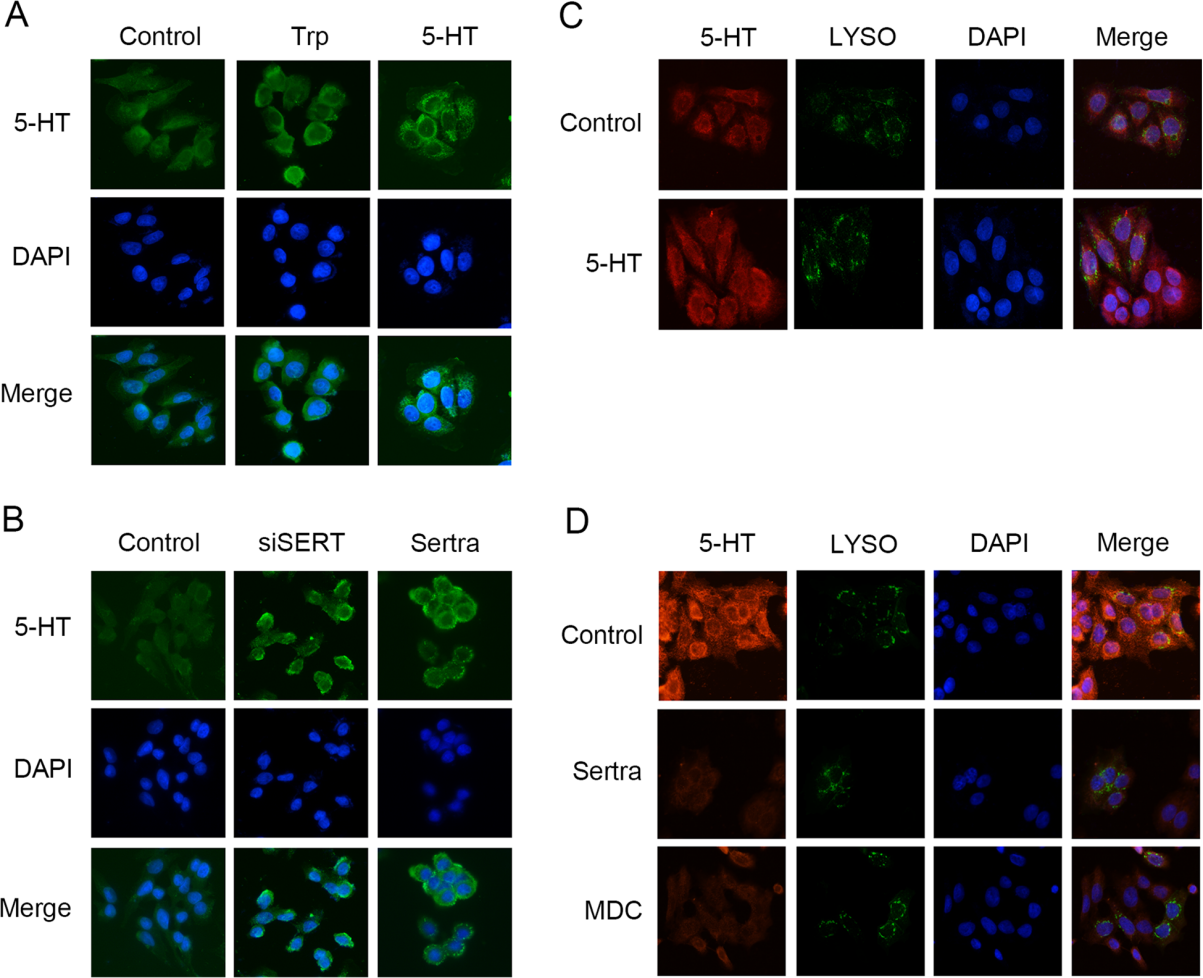
Endogenous and exogenous serotonin have different subcellular localizations. **a** SW480 cells were starved with Trp-free and serum-free medium for 24 h after which they were supplied with Trp (75 μM, 9 h) or serotonin (50 μM, 4 h), respectively. Serotonin localization was visualized by IF staining with anti-serotonin (green). DNA was stained with DAPI (blue). Scale bar: 25 μm. **b** SW480 cells were transfected with siSERT (48 h) or negative control (48 h) or sertraline (15 μM, 12 h). Serotonin localization was visualized by IF staining with anti-serotonin (green). DNA was stained with DAPI (blue). Scale bar: 25 μm. **c** SW480 cells were transfected with lyso-EGFP (12 h) or the negative control (12 h) and starved with FBS-free medium (24 h) followed by serotonin (50 μM) stimulation (4 h) . Serotonin localization was visualized by IF staining with anti-serotonin (red) while lysosomal localization was visualized as green. DNA was stained with DAPI (blue). Scale bar: 25 μm. **d** SW480 cells were transfected with lyso-EGFP (24 h) or negative control (24 h) and then treated with sertraline (15 μM) or MDC (200 μM) for 30 min. Serotonin localization was visualized by IF staining with anti-serotonin (red) while lysosome localization was visualized as green. DNA was stained with DAPI (blue). Scale bar: 25 μm

The above results indicate that SERT inhibition decreases intracellular serotonin levels to suppress mTOR serotonylation and activity. However, this postulation contradicted our previous finding that SERT inhibition elevated intracellular serotonin levels. Therefore, we postulated that endogenous and exogenous serotonin have different subcellular localizations at the early stage, and mTOR is more vulnerable to exogenous serotonin-mediated serotonylation. Immunofluorescence showed that endogenous serotonin, synthesized from Trp, was mainly localized around the nucleus, while exogenous serotonin, transported by SERT, was widely distributed at the periphery of the cytoplasm (Fig. 5a, Additional file 6: Figure S6A). SERT inhibition by siRNA or sertraline treatment resulted in elevated levels of serotonin around the nucleus (Fig. 5b, Additional file 6: Figure S6B). Membrane SERT enters cells through endocytosis [21]. Therefore, we hypothesized that exogenous serotonin might enter cells through the endosome-lysosome pathway. Subsequently, we investigated serotonin co-localization with lysosomes and found that, compared to endogenous serotonin, exogenous serotonin exhibited better co-localization with lysosomes, which could be reversed by sertraline and MDC treatment (Fig. 5c, d). Since lysosome was the site of mTORC1 activation. We hypothesized that mTORC1 plays a role in intracellular serotonin concentration changes and regulates the balance of endogenous and exogenous serotonin pathway. In conclusion, our results suggest that SERT inhibition suppresses serotonylated modification of mTOR by reducing serotonin concentrations in the lysosomes.

### Enhancing Trp metabolism promotes colon cancer growth

**Fig. 6:**
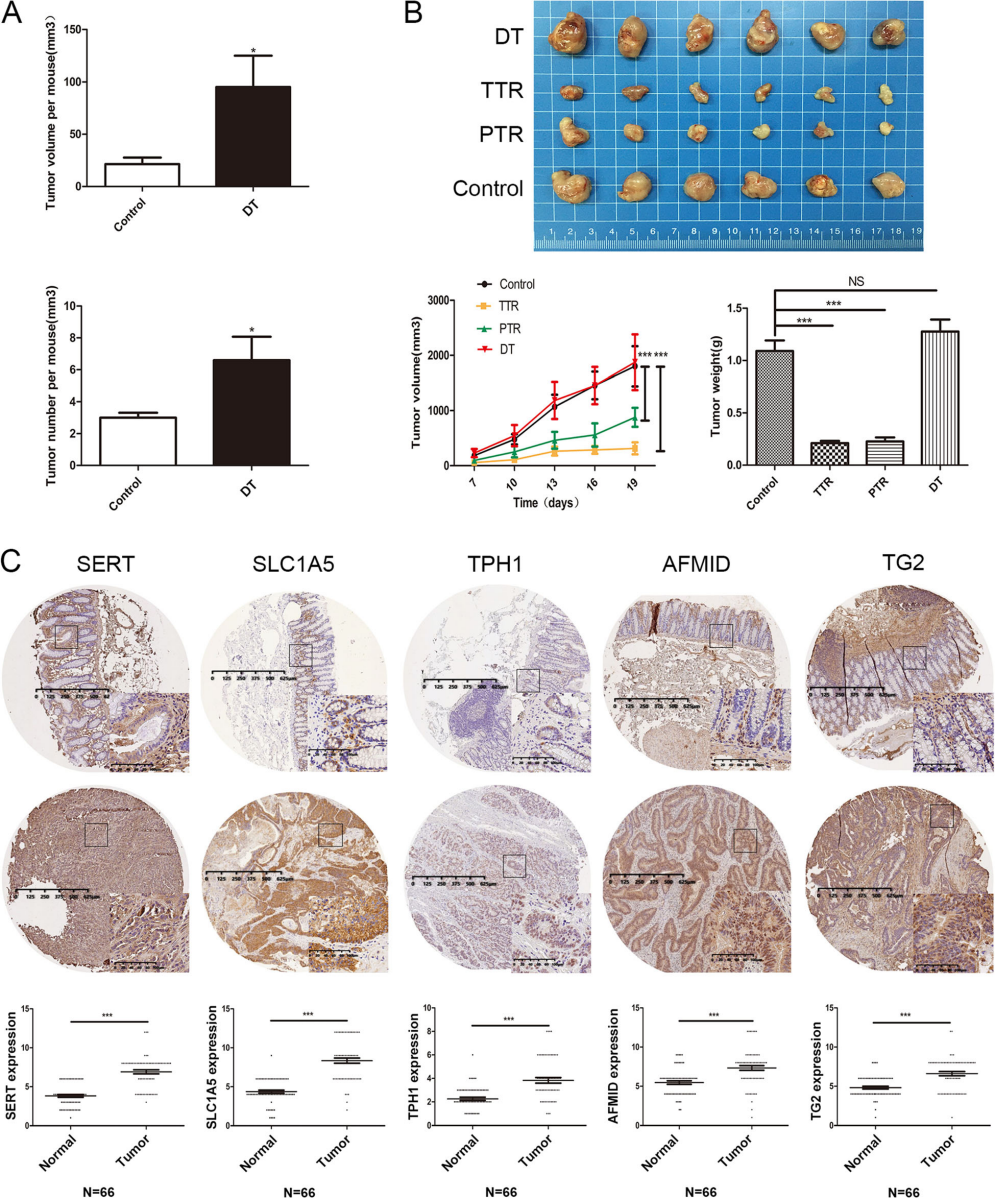
Effect of enhancing or limiting tryptophan metabolism in colon cancer mice models. **a** The linear graph shows the average number and tumor area for control group and DT group (mean ± SD). **b** CT-26 homografts were established in 6-8-week old female BALB/c mice. Once tumors were palpable, mice were randomized into groups fed on a DT diet (4 mg/kg Trp, *n* = 6), total tryptophan restriction (TTR) diet (0 mg/kg Trp, *n* = 6), partial tryptophan restriction (PTR) diet (0.6 mg/kg Trp, *n* = 6), or a control diet (2 mg/kg Trp, *n* = 6). Tumor volumes and mice weights were monitored over time. At the end of the experiment, tumors were dissected, imaged and weighed. The bar graph displays the tumour volume and weight of each group (mean ± SD). Statistical methods: one-way ANOVA, *p < 0.05, **p < 0.01, ***p < 0.001, NS indicates no significant difference. **c** Representative images of Trp metabolism-related proteins in clinical samples of colon cancer tissues and normal colon tissues. IHC analysis of SERT, SLC1A5, TPH1, AFMID, and TGM2 expression in clinical samples of colon cancer tissues and normal colon tissues. The method assigning IHC score for each sample was described in “Materials And Methods” section. *** *p* < 0.001

To determine the role of enhanced Trp metabolism in SERT-targeted (SSRIs) colon cancer treatment, we established a AOM/DSS-induced colon cancer mice models. Mice were fed on diets containing different amounts of tryptophan. Compared to the control diet, the double tryptophan (DT) diet increased the average tumor volume and number (Figs. 2d, 6a). All groups consumed comparable amounts of food, implying that the effect was not due to caloric intake. Further investigations revealed that tryptophan restriction inhibited the growth of the subcutaneous homograft tumor model with CT-26 (Fig. 6b). Given that the total tryptophan restriction (TTR) group mice survived severe weight loss, completely limiting Try intake might not be suitable. However, in the partial tryptophan restriction (PTR) group, mice did not exhibit significant changes in weight and food intake, compared to the control group. Therefore, the PTR diet was applied in our experiments. In vitro, exogenous Trp addition promoted the proliferation of HCT116 and SW480 colon cancer cells (Additional file 7: Figure S7A). Taken together, the above data indicate that enhanced Trp metabolism can promote colon cancer tumor growth.

### High expression levels of Trp and serotonin metabolism-related enzymes in colon cancers

To characterize the Trp metabolic pathway in colon cancer tissues, expressions of Trp transporters (SLC1A5 and SLC7A5) and Kyn metabolism-related enzymes (IDO1 and AFMID) as well as serotonin metabolism-related enzymes (TPH1, MAOA, MAOB and TG2) were analyzed in a colon cancer tissue microarray (TMA) containing 66 matched pairs of carcinoma and adjacent tissue samples by immunohistochemistry (IHC). Compared to normal tissues, all enzymes were over expressed in colon cancer tissues (Fig. 6c, Additional file 8: Figure S8A). Collectively, our results show that Trp metabolism is enhanced in colon cancer, suggesting that blocking the Trp metabolic pathway is a potential target in colon cancer treatment.

### Anti-tumor effects of a combination of SERT inhibition and dietary Trp restriction or trametinib in vitro and in vivo

**Fig. 7:**
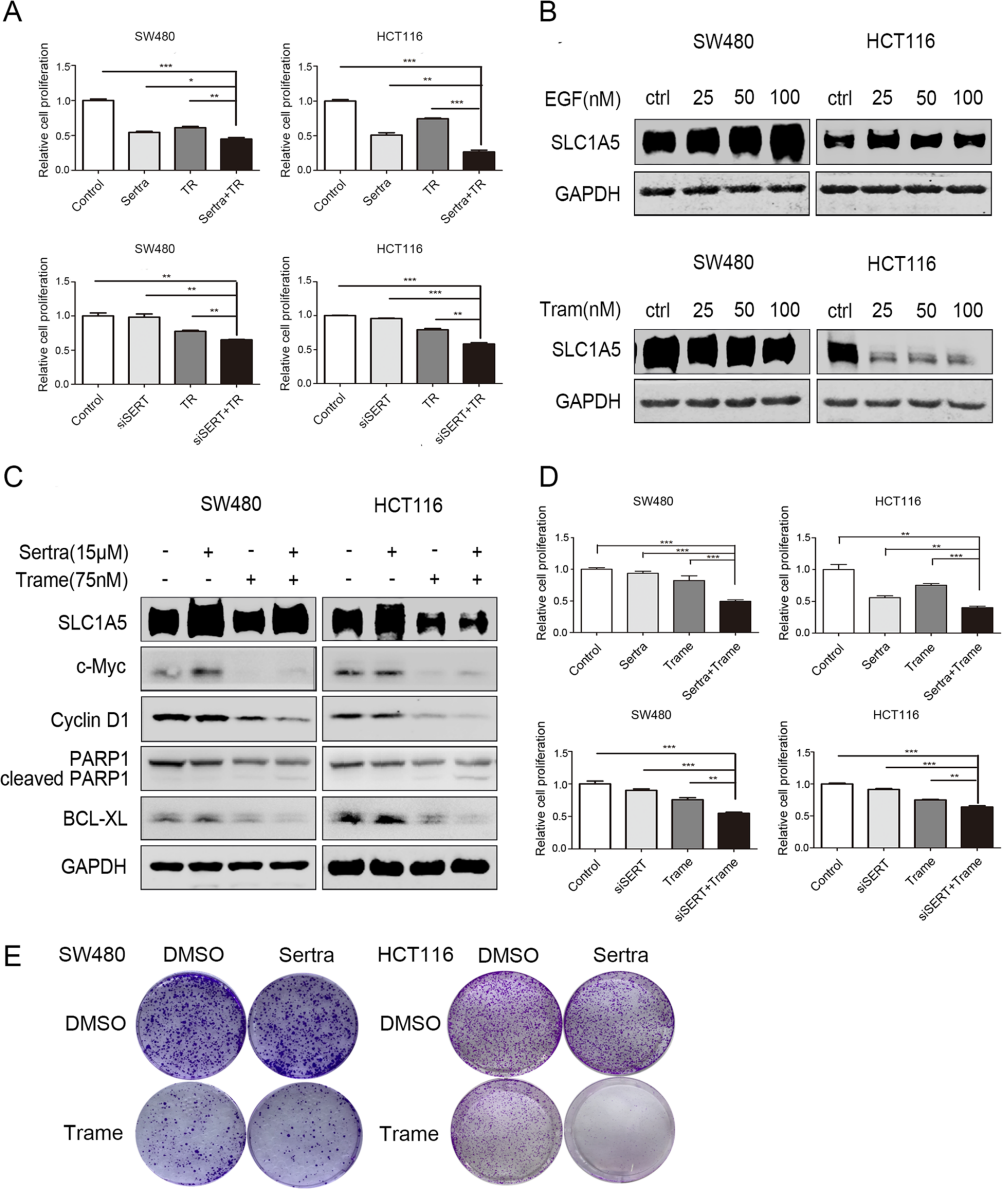
Anti-tumor effect of a combination SERT inhibition with dietary trp restriction or trametinib in vitro*.* **a** Proliferation assays using the Cell Counting Kit-8 in SW480 and HCT116 cells transfected with siSERT or treated with sertraline in the presence or absence of Trp (75 uM) at day 2. Data were presented as the mean ± SD. **b** Colon cancer cells (SW480 and HCT116) were starved in serum-free medium for 24 h and stimulated with EGF (25-100 nM) for another 24 h or directly treated with trametinib (25-100 nM) for 24 h, after which WB analysis of SLC1A5 was performed. **c** WB for SLC1A5, c-Myc, Cyclin D1, cleaved PARP and BCL-XL in SW480 and HCT116 cells treated with 15 μM sertraline with or without trametinib (75 nM). **d** Proliferation assays using the Cell Counting Kit-8 in SW480 and HCT116 cells transfected with siSERT or treated with sertraline in the presence or absence of trametinib at day 2. Data are presented as the mean ± SD. **e** SW480 and HCT116 cells were incubated with DMSO, sertraline (2 μM), trametinib (25 nM) and combined treatment for 7 d, respectively. Cell proliferation abilities were evaluated by the colony formation assay

Studies have identified SSRIs as promising therapeutic options in cancer treatment, however, the above findings suggest that enhanced Trp metabolism may weaken the anti-tumor activity of SSRIs. Silencing SERT slightly inhibited HCT116 cell proliferations, but did not affect SW480 proliferation (Additional file 9: Figure S9A). In SW480 and HCT116 cells, SSRIs exhibited more effective cell proliferation inhibitions than SERT silencing, and they still exerted inhibitory effects after SERT silencing, indicating that SSRIs inhibit additional targets to suppress cell proliferation (Additional file 9: Figure S9B, C). However, in the absence of Trp, cell proliferations were inhibited by SERT interference or sertraline treatment (Fig. 7a).

**Fig. 8:**
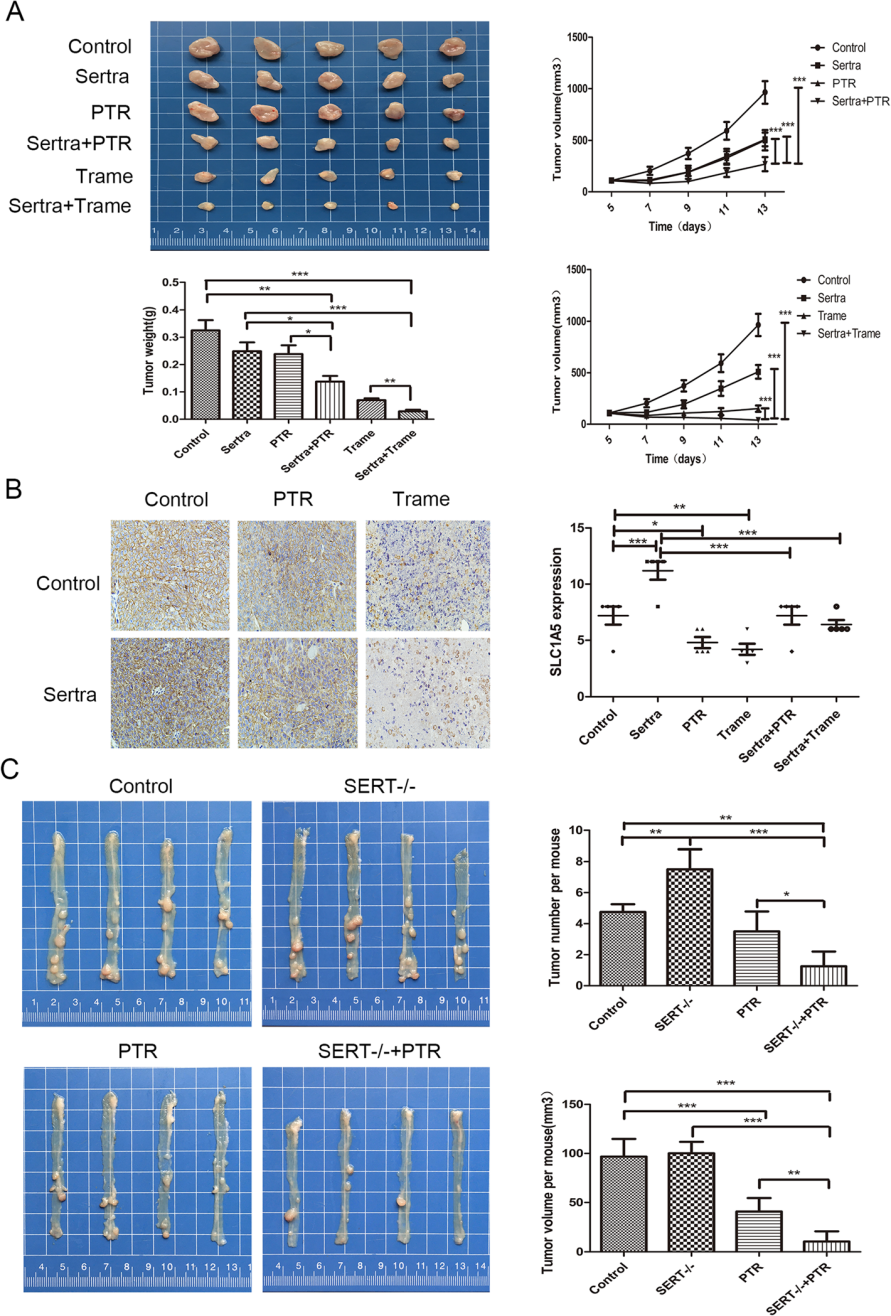
Anti-tumor effect of a combination SERT inhibition with dietary trp restriction or trametinib in vivo*.* **a** Subcutaneous tumors were formed in nude mice injected with HCT116. Once the tumors were palpable, mice were randomly distributed into six groups (*n* = 5) including the control group (DMSO), sertraline group (30 mg/kg), trametinib group (2 mg/kg), PTR group, sertraline plus PTR group and sertraline plus trametinib group. The diet for all mice were switched for PTR or specific control diet, 1 week before injection. The bar graph shows the tumor weights for each group (*n* = 5). Tumor sizes and mice weights were measured after every 2 days. Line charts show the tumor volumes for each group (*n* = 5). Data are presented as the mean ± SD. **b** Representative pictures of SLC1A5 expression in xenograft tumor tissues. The bar graph indicates the IHC scores of SLC1A5 expression. **c** Mouse model of AOM/DSS-induced colon cancer was established in SERT-KO and SERT-WT mice. One month after AOM injection, the mice were randomly divided into 4 groups (*n* = 4) including the control group, SERT-KO group, PTR group and SERT-KO plus PTR group. All mice were fed with PTR diet and matched control diet during the period of drinking normal water. After 14 weeks, tumors were dissected and their volumes and number determined. The graphs showed the average tumor number and volume of each group. Statistical methods: one-way ANOVA followed by LSD post hoc test, *p < 0.05, **p < 0.01, ***p < 0.001

Trametinib, a small-molecule inhibitor that targets MEK, had an inhibitory effect on the transportation of multiple amino acids. Transportation of tryptophan, induced by EGF stimulation, and SCL1A5 up-regulation, induced by sertraline, were significantly reversed by trametinib treatment (Fig. 7b, c). The CCK-8 and colony formation assays showed that sertraline synergized with Trp restriction and trametinib to inhibit cell viabilities of HCT116 and SW480 cells (Fig. 7d, e, Additional file 10: Figure S10A). To validate the synergistic effect in vivo, we constructed a mouse xenograft model of HCT116 cells. The results revealed that sertraline could combine with either dietary Trp restriction or trametinib to suppress tumor growth (Fig. 8a). There were no significant differences in body weights and food intake between the different groups. IHC analysis showed that the combination of sertraline and trametinib significantly suppressed the expression levels of SLC1A5 in the tumors (Fig. 8b). When we applied PTR diet on the basis of SERT deficiency in AOM/DSS-induced colon cancer mouse model, PTR diet reversed the tumorigenic promotion and synergized with of SERT deficiency in suppressing the formation and growth of the tumor (Fig. 8c). In summary, a combination of sertraline and dietary trp restriction or trametinib showed synergistic anti-tumor activities in vitro and in vivo*.*

## Discussion

Intracellular serotonin has two main sources including endogenous synthesis from Trp and exogenous uptake via SERT. Uptake of Trp in the intestines is done by enteroendocrine units using an amino acid transport system including SLC1A5 and SLC7A5. Endogenous serotonin is synthesized from Trp through TPH. There are 2 isoforms of TPH enzymes that are involved in serotonin biosynthesis. Most of the peripheral serotonin are synthesized through TPH1 in the intestines, whereas TPH2 is predominantly present in the cerebrocentric and myenteric plexus neurons [1, 22]. Once synthesized, serotonin is packaged into intracellular vesicles by the vesicular monoamine transporter (VMAT).

Under physiological conditions, most of the cellular serotonin is transported by SERT from extracellular spaces and is sufficient to maintain physiological functions, while endogenous serotonin synthesis is commonly inactive. Trp biodegradation and subsequent serotonin biosynthesis mainly occurs in the mitochondrial outer membrane. VMAT1 transfers the newly synthesized serotonin from the cytosol to storage vesicles before its release. After its release and activation, serotonin is reabsorbed by SERT. Intracellular serotonin functions through serotonylation or is restored by VMAT1 for the next release. Serotonin can be degraded by monoamine oxidase (MAO)-mediated oxidative deamination, producing 5-HIAA in the mitochondrial outer membrane.

In the intestines, Trp metabolism occurs through three main pathways, all of which are widely indicated in cancer: i. The kynurenine pathway (KP); ii. The serotonin pathway, and iii. The protein synthesis pathway. Kyn production is catalyzed by three enzymes: IDO1, IDO2, and TDO2 and is finally transformed into Kyn by arylformamidase (AFMID). In this study, we found that inhibiting exogenous serotonin uptake promoted Trp metabolism, which in turn produced serotonin. The underlying mechanism is that the decreased level of intracellular serotonin inhibited mTOR activity, which then upregulated the SLC1A5 protein levels, enhancing Trp metabolism and serotonin biosynthesis. We identified a novel feedback mechanism through which cells maintain intracellular serotonin metabolism homeostasis in the absence of SERT. Exogenous and endogenous serotonin are likely to have diverse subcellular locations at least before redistribution, and may be the reason why intracellular serotonin concentrations may be higher than its original level after sertraline treatment.

Genetically or pharmacologically inhibiting SERT decreased intracellular serotonin concentrations, resulting in the reduction of mTOR serotonylation and activity. MTOR is a vital energy sensor that is involved in the metabolic regulation of various substances [23]. Therefore, a decrease in mTOR activity promotes Trp absorption and its subsequent catabolism to maintain intracellular serotonin levels. Our results demonstrate that inhibiting serotonin reuptake by blocking SERT in CC leads to increased absorption and processing of Trp both in vivo and in vitro. Although we were unable to detect metabolite levels in the tumor due to their limited quantities in mice, changes in plasma metabolite levels also have a reference value. Compared to the control group, SERT-WT mice treated with sertraline or SERT-KO mice exhibited decreased Trp abundance and increased Kyn levels in serum.

Serum serotonin levels in tumor mice (AOM/DSS group) were elevated than those in the blank group (Additional file 11: Figure S11). Interestingly, we also detected extremely low serum serotonin levels in SERT-KO mice (Additional file 11: Figure S11). It is possible that serotonin metabolic pathway in the intestines of SERT-KO mice was impaired by blocked inactivation, thereby reducing secretion. However, this finding contrasts with that of previous studies, which reported that serotonin is found in all normal sites in the gut of SERTKO mice as well as physiologic number of serotonin-producing enterochromaffin cells.

Additionally immunohistochemical detection of colon cancer micro array revealed that the expression of serotonin metabolism-related enzymes (TPH1, SERT, MAOA, and MAOB) were overexpressed in human colon cancer tissues than in the adjacent normal tissues. These findings suggest that tumor cells have an increased demand for, and actively metabolize serotonin. When SERT is deleted, cells have to increase endogenous serotonin biosynthesis. It was shown that TPH expression is upregulated in the gut of SERTKO mice, while the absorption of carbohydrates, fats and peptides were also elevated [24, 25], suggesting that SERT-KO mice have metabolic disorders that may promote tumorigenesis under the induction of carcinogenic factors.

Serotonin confers its biological functions in receptor-dependent or receptor-independent modes. It binds its receptors (5-HTR) to trigger a number of events through complex molecular mechanisms. Conventionally, the serotonin that is taken up SERT is for recycling or degradation, however, a few studies have elucidate on its intracellular functions. Cellular serotonin has been shown to form covalent bonds with cytosolic proteins such as small GTPases, RhoA and Rab by transglutaminase 2 (TG2) through transamidation and altered signaling properties of mono-aminylated substrates, a process referred to as serotonylation.

Serotonylation is involved in the pathogenesis of various diseases, including pulmonary hypertension and diabetes [10, 26]. This process depends on intracellular serotonin levels and is sensitive to pharmacological inhibition of TG2. TG2 is highly expressed in human colon cancer tissues, and is a potential prognostic marker [27]. Histone (H3K4me3) is a predictive factor for poor clinical outcomes in colon cancer, and may also be an endogenous serotonin substrate [28]. Therefore, serotonylation is likely to also play a vital role in tumor development. The use of rapamycin to treat cells over-expressing TG was shown to overcome tTG-induced survival outcomes [29], and promoted a compensatory increase in transglutaminase 2 (TG2) levels in mTORC1-driven tumors, thereby limiting rapamycin effectivity [30]. In view of the role of TG2 in serotonylation, TG2 is likely to play an important role in serotonin-induced mTORC1 activation.

In our findings, elevated serotonylated mTOR levels were detected after serotonin treatment, and were reversed by siTG2, MDC, or sertraline treatment. Genetic silencing and early pharmacologic inhibitions of SERT partly eliminated mTORC1 activation, which supported the concept that SERT is involved in serotonin-driven mTOR. Serotonin mediates PDAC and HCC proliferation through the activation of mTOR signaling by targeting HTR2B [31, 32]. 5-HTRs signaling leads to Ca 2+ release, which activates TG2 [26]. To verify whether 5-HTRs are involved in this process, we treated cells with the broad-spectrum 5-HTRs inhibitor, Asenapine. Asenapine did not abolish serotonin-induced mTORC1/p70s6k pathway activation. These findings indicate that serotonin activates mTORC1 through serotonylation, rather than through a receptor-dependent manner.

SERT targeted drugs have anti-tumor activities [33], however, their underlying mechanisms have not been established. SSRIs exert anti-cancer effects in a variety of ways. Fluoxetine inhibits tumor multidrug resistance by regulating the expressions of P-glycoprotein (P-gp), ABCB1 (MRP1), and glutathione S-transferase-pi (GST-π), which sensitizes tumors to chemotherapy [34–36]. The same effect has been reported in sertraline [37]. Sertraline can also inhibit the formation of breast tumor initiating cells (BTIC), promoting tumor occurrence and development and inducing tumor resistance to radiotherapy and chemotherapy [38]. Di Rosso et al. found that fluoxetine and sertraline could regulate immunity and restore the significant reduction in anti-tumor immune responses induced by chronic stress [39]. In addition, sertraline can regulate autophagy through the AMPK/mTOR pathway, which was found to inhibit the effect of erlotinib in the treatment of non-small cell lung cancer [40]. Sertraline can also induce apoptosis and autophagy in prostate cancer stem cells (PCSC) [41].

We found that SERT knockout and pharmacological inhibition of SERT exerted diverse effects on tumor development in colon cancer mouse models. The accelerative effect of SERT deficiency on colon cancer might due to increased Trp uptake and metabolism, while sertraline may act by suppressing other targets to inhibit tumor growth. Promotion of Trp metabolism enhances the occurrence and development of colon cancer. We confirmed that the main transporters and metabolic enzymes in the Trp metabolism pathway are highly expressed in colon cancer. The PTR diet or trametinib treatment reduced Trp uptake and degradation, and enhanced the anti-tumor activity of sertraline in vivo and in vitro.

Our results indicate that inhibiting exogenous serotonin uptake may not be effective in colon cancer treatment, therefore, targeting both exogenous and endogenous 5-HT pathways may be a better choice. In the United States, sertraline, a representative serotonin selective reuptake inhibitor, was approved as a clinical antidepressant with appropriate biocompatibilities [42], its safety and effectiveness are guaranteed. Epidemiological studies have reported that SSRIs reduce the risks of various tumors and are promising drugs in tumor treatment.

## Conclusions

In our study, the results of in vitro and in vivo experiments elucidate on SERT-targeted therapy of colon cancer, showing a novel feedback mechanism by which cancer cells maintain the homeostasis of intracellular serotonin, and suggesting a novel SSRIs-based strategy for colon cancer treatment.

## Supplementary Information

**Additional file 1: Figure S1.** The effect of SERT interference or inhibition on expression of key enzymes in the catabolic pathway of tryptophan. A RT-PCR for SERT, SLC1A5, SLC7A5, TDO2, AFMID and TPHI mRNA expression levels in HCT116 cells transfected with siSERT or negative control (48 h). mRNA expressions were normalized to GAPDH. B RT-PCR for SLC1A5, SLC7A5, TDO2, AFMID and TPHI mRNA expressions in HCT116 cells treated with sertraline (15 μM, 12 h) or DMSO. mRNA expression levels were normalized to GAPDH. *p < 0.05, **p < 0.01, ***p < 0.01, using the Student’s t test (two-tailed).

**Additional file 2: Figure S2.** The effect of serotonin and kyn on the proliferation of colon cancer cells. A,B Proliferation assays using the Cell Counting Kit-8 in SW480 and HCT116 cells treated with increasing concentrations of serotonin (1-100 μM) and Kyn (5-100 μM) at day 2. Data are presented as the mean ± SD, *p < 0.05, **p < 0.01, using the Student’s t test (two-tailed).

**Additional file 3: Figure S3.** The Mrna expression of the indicated Trp transporters and enzymes in normal colon tissues. A RT-PCR for Mrna expression of the indicated Trp transporters and enzymes in normal colon tissues of SERT-WT and SERT-KO mice. Gene expression was normalized to GAPDH.

**Additional file 4: Figure S4.** Inhibition of SERT suppresses activation of Mtor by inhibiting serotonin reuptake. A, B WB for Mtor, S6K, SLC1A5 and SERT using SW480 and HCT116 cells treated with increasing concentrations of MHY1485 (25-100 μM) or Rapamycin (25-100 nM). C SW480 and HCT116 cells were transfected with negative control siRNA or SERT siRNA for 24 h, then starved in serum-free medium for 24 h, and incubated with DMSO or serotonin (100 μM) for another 12 h. The expression of SERT, S6K, and SLC1A5 was detected by western blot; D SW480 and HCT116 cells were treated with DMSO or sertraline (15 μM) for 12 h with or without serotonin (100 μM). Expression levels of SERT, S6K and SLC1A5 were detected by western blot.

**Additional file 5: Figure S5.** Serotonin receptor broad-spectrum inhibitors cannot inhibit serotonin activation of Mtor. A WB for mTOR, S6K, SERT and SLC1A5 using SW480 and HCT116 cells treated with increasing concentrations of Asenapine (1-100 μM).

**Additional file 6: Figure S6.** Endogenous and exogenous serotonin have different subcellular locations. A HCT116 cells were starved with Trp-free and serum-free medium for 24 h after which they were supplied with Trp (75 μM, 9 h) or serotonin (50 μM, 4 h) for 9 h, respectively. Serotonin was demonstrated by anti-serotonin staining (green). DNA was stained with DAPI (blue). Scale bar: 25 μm. B HCT116 cells were transfected with siSERT (48 h) or negative control (48 h) or sertraline (15 μM, 12 h). Serotonin localization was visualized by IF staining with anti-serotonin (green). DNA was stained with DAPI (blue). Scale bar: 25 μm.

**Additional file 7: Figure S7.** The effect of tryptophan on the proliferation of colon cancer cells. A SW480 and HCT116 cells were cultured in 96-well plates in DMEM containing 10% FBS and incubated overnight. The next day, cells were starved with Trp-depletion and serum-free medium for 24 h. Thereafter, cells were cultured in a medium supplemented with Trp (10-100 μM) or without Trp for an additional 2 days. Then, their viability was detected using a CCK8 kit.

**Additional file 8: Figure S8.** Expression of SLC7A5, IDO1, MAOA and MAOB in colon cancer tissues. A Representative images of Trp metabolism-related proteins in clinical samples of colon cancer and normal colon tissues. B: IHC analysis of SLC7A5, IDO1, MAOA and MAOB expression in clinical samples of colon cancer tissues and normal colon tissues. The method for assigning IHC scores for each sample was described in the “Materials and methods” section. *** *p* < 0.001.

**Additional file 9: Figure S9.** The effect of silencing and inhibiting SERT on the proliferation of colon cancer cells. A Proliferation assays for SW480 and HCT116 cells transfected with siSERT were performed using the Cell Counting Kit-8 at day 3. B Proliferation assays for SW480 and HCT116 cells treated with increasing concentration of sertraline (5-15 μM) at day 2. C SW480 and HCT116 cells were transfected with siSERT for 24 h and treated with sertraline (10 μM) for another 48 h. The Cell Counting Kit-8 experiment was used to detect cell vitality. Data presented are presented as the mean ± SD, *p < 0.05, **p < 0.01, ***p < 0.001 using the Student’s t test (two-tailed).

**Additional file 10: Figure S10.** Effect of trametinib on the proliferation of colon cancer cells. A Proliferation assays were performed on SW480 and HCT116 cells treated with increasing concentrations of trametinib (25-100 nM) at day 2. Data are presented as the mean ± SD, *p < 0.05, **p < 0.01, ***p < 0.001 using Student’s t test (two-tailed).

**Additional file 11: Figure S11.** The effect of knocking-out SERT on plasma serotonin levels in normal mice and in colon cancer mouse model. Serum levels of serotonin in SERT-WT and SERT-KO mice in normal and AOM/DSS group were detected using LCMS/MS.

**Additional file 12: Table S1.** Primer sequence.

## Data Availability

The datasets supporting the conclusions of this article are included within the article and its additional files.

## Abbreviations

SERT: Serotonin transporter
Trp: Tryptophan
SSRIs: Selective serotonin reuptake inhibitors
5-HT: 5-hydroxytryptamine
HTR: Serotonin receptors
TG2: Transglutaminase 2
TPH: Tryptophan hydroxylase
EAA: Essential amino acid
SLC1A5: Solute carrier 1A5
SLC7A5: Solute carrier 7A5
AOM: Azoxymethane
DSS: Dextran sodium sulfate
VMAT: Vesicular monoamine transporter
MAO: Monoamine oxidase
KP: Kynurenine pathway
