# Supplementary figures and images for: Targeting SERT promotes tryptophan metabolism: mechanisms and implications in colon cancer treatment
Source: J Exp Clin Cancer Res. 2021 May 18;40:173. doi: 10.1186/s13046-021-01971-1 (PMC8132442; doi:10.1186/s13046-021-01971-1)

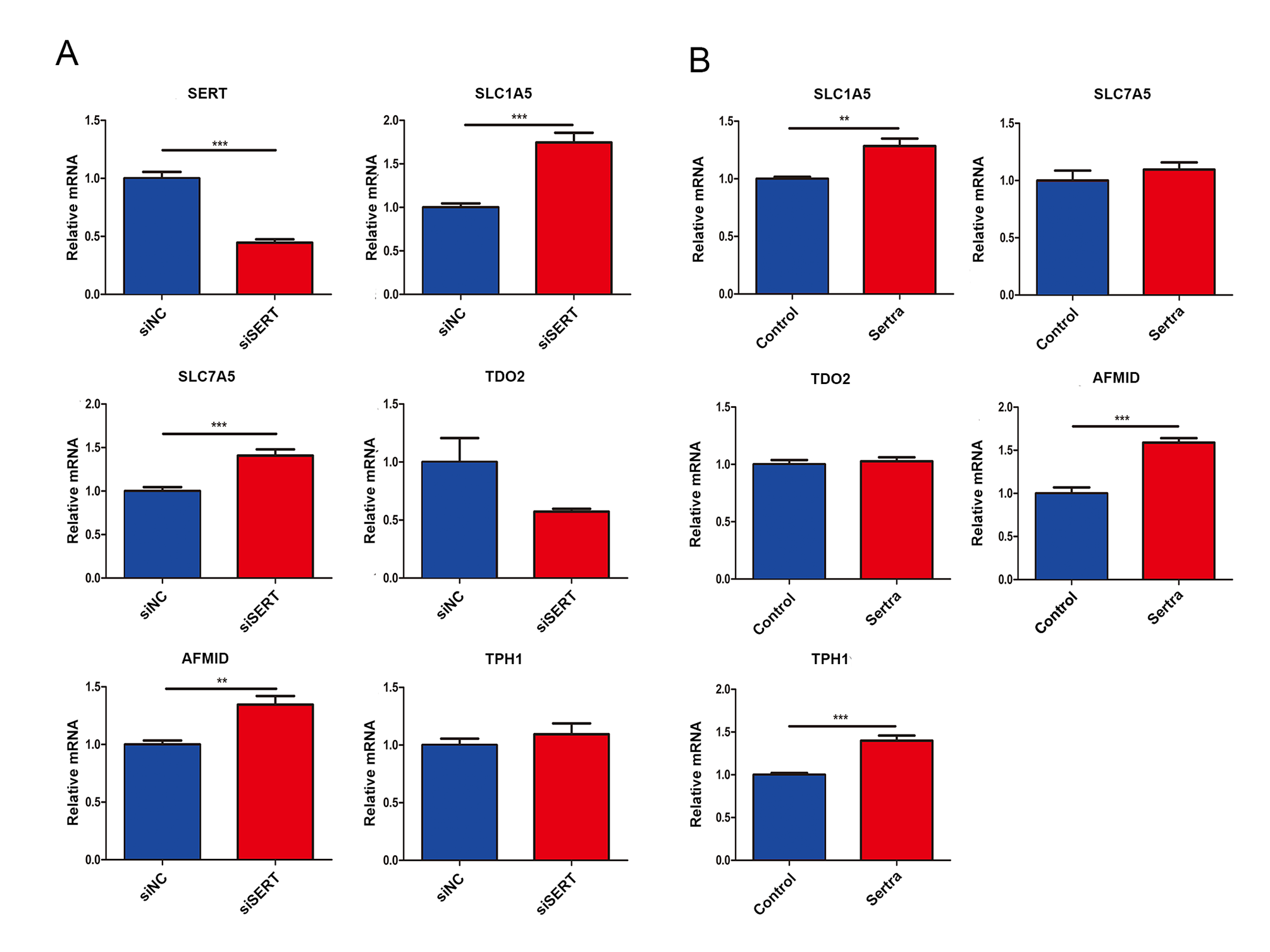

Supplement: Supplementary file 1 — Additional file 1: Figure S1. The effect of SERT interference or inhibition on expression of key enzymes in the catabolic pathway of tryptophan. A RT-PCR for SERT, SLC1A5, SLC7A5, TDO2, AFMID and TPHI mRNA expression levels in HCT116 cells transfected with siSERT or negative control (48 h). mRNA expressions were normalized to GAPDH. B RT-PCR for SLC1A5, SLC7A5, TDO2, AFMID and TPHI mRNA expressions in HCT116 cells treated with sertraline (15 μM, 12 h) or DMSO. mRNA expression levels were normalized to GAPDH. *p < 0.05, **p < 0.01, ***p < 0.01, using the Student’s t test (two-tailed). [file 13046_2021_1971_MOESM1_ESM.tif]

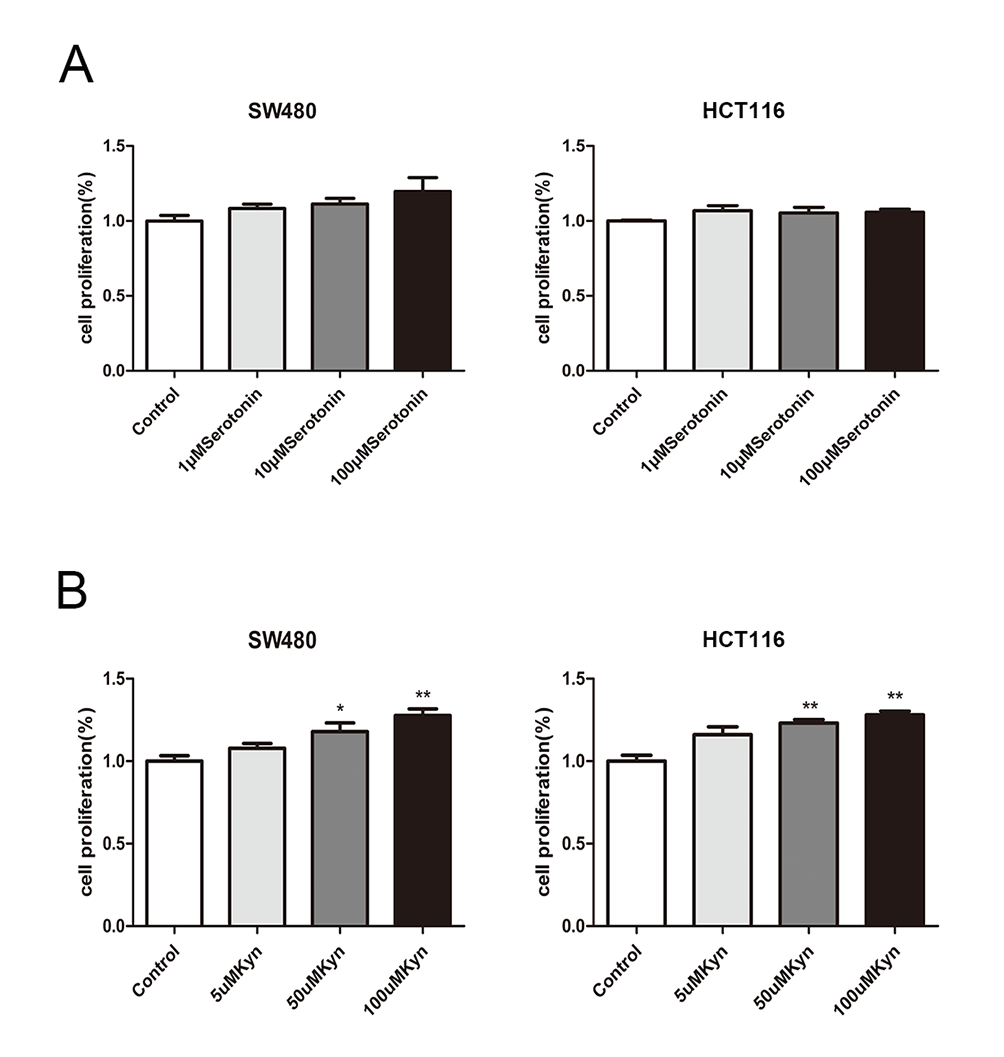

Supplement: Supplementary file 2 — Additional file 2: Figure S2. The effect of serotonin and kyn on the proliferation of colon cancer cells. A,B Proliferation assays using the Cell Counting Kit-8 in SW480 and HCT116 cells treated with increasing concentrations of serotonin (1-100 μM) and Kyn (5-100 μM) at day 2. Data are presented as the mean ± SD, *p < 0.05, **p < 0.01, using the Student’s t test (two-tailed). [file 13046_2021_1971_MOESM2_ESM.tif]

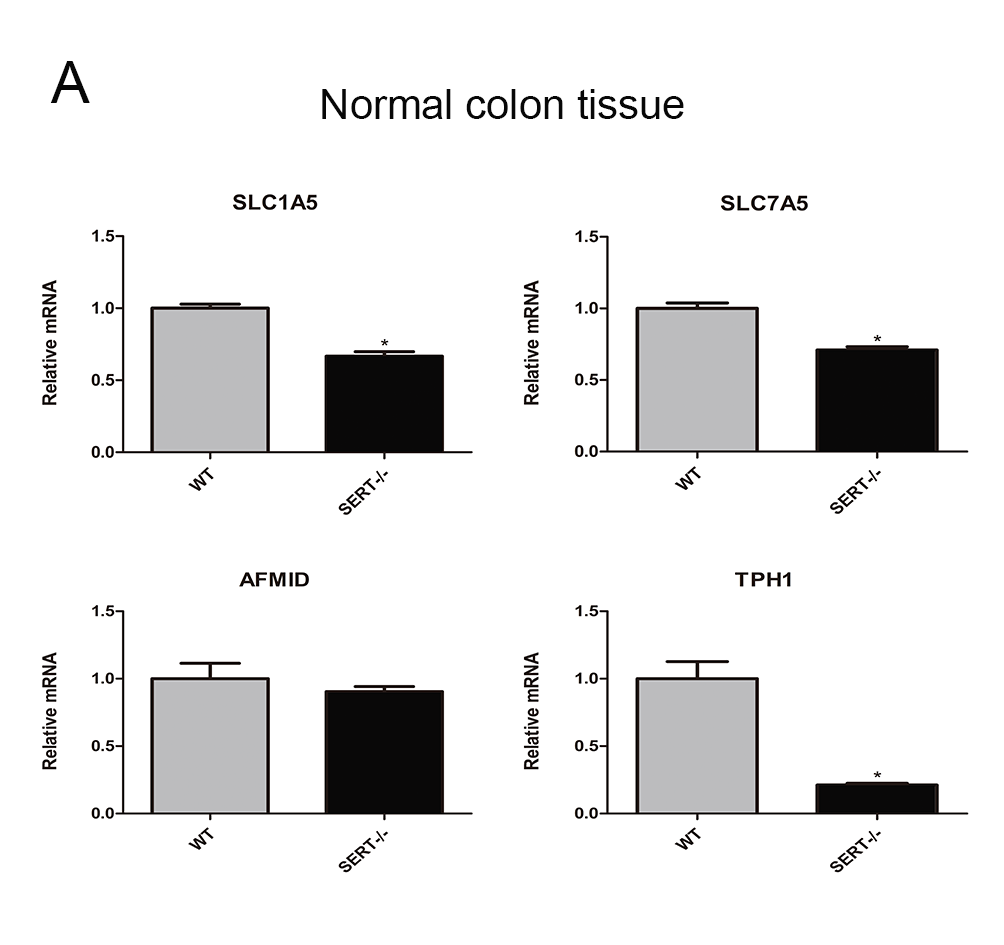

Supplement: Supplementary file 3 — Additional file 3: Figure S3. The Mrna expression of the indicated Trp transporters and enzymes in normal colon tissues. A RT-PCR for Mrna expression of the indicated Trp transporters and enzymes in normal colon tissues of SERT-WT and SERT-KO mice. Gene expression was normalized to GAPDH. [file 13046_2021_1971_MOESM3_ESM.tif]

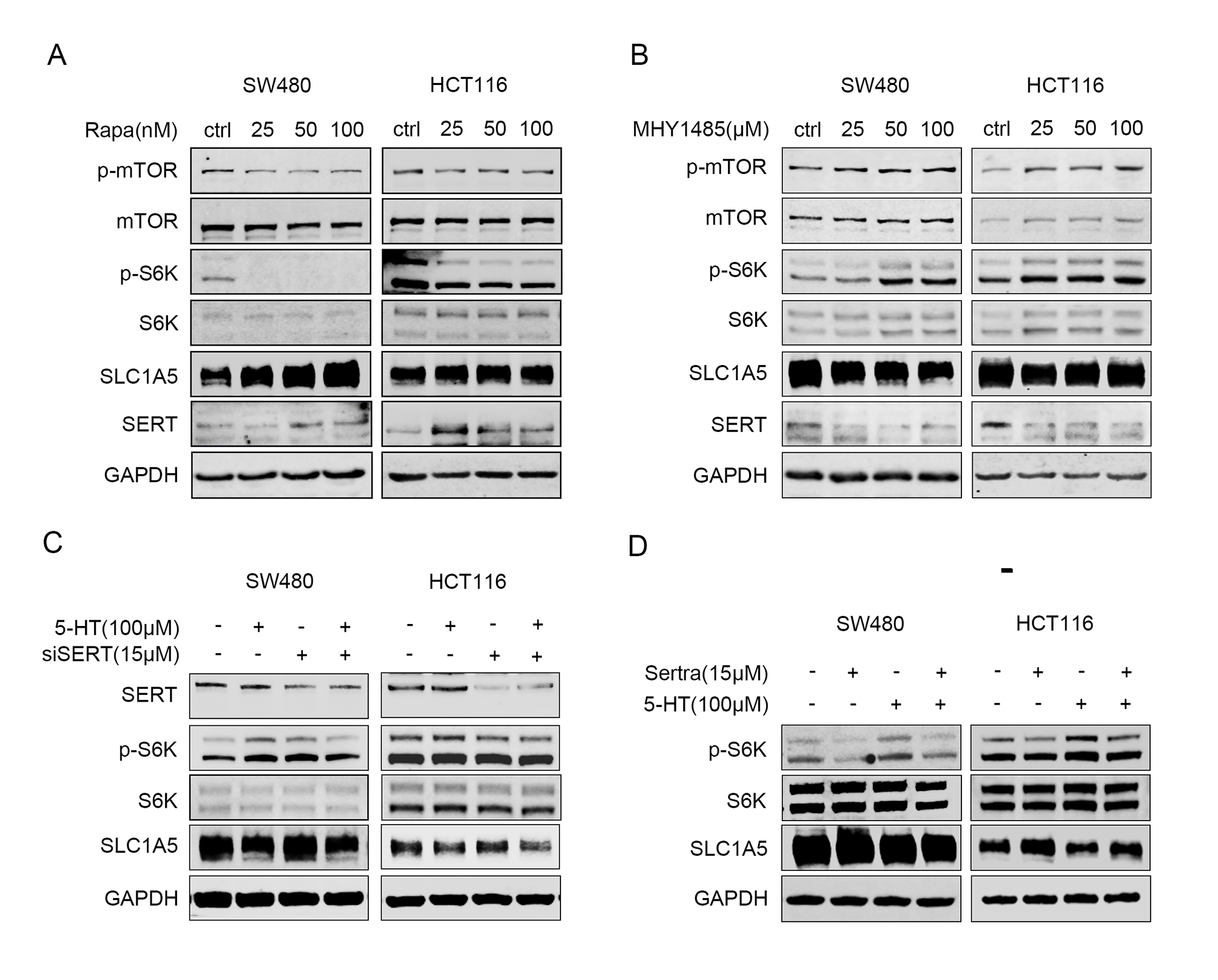

Supplement: Supplementary file 4 — Additional file 4: Figure S4. Inhibition of SERT suppresses activation of Mtor by inhibiting serotonin reuptake. A, B WB for Mtor, S6K, SLC1A5 and SERT using SW480 and HCT116 cells treated with increasing concentrations of MHY1485 (25-100 μM) or Rapamycin (25-100 nM). C SW480 and HCT116 cells were transfected with negative control siRNA or SERT siRNA for 24 h, then starved in serum-free medium for 24 h, and incubated with DMSO or serotonin (100 μM) for another 12 h. The expression of SERT, S6K, and SLC1A5 was detected by western blot; D SW480 and HCT116 cells were treated with DMSO or sertraline (15 μM) for 12 h with or without serotonin (100 μM). Expression levels of SERT, S6K and SLC1A5 were detected by western blot. [file 13046_2021_1971_MOESM4_ESM.tif]

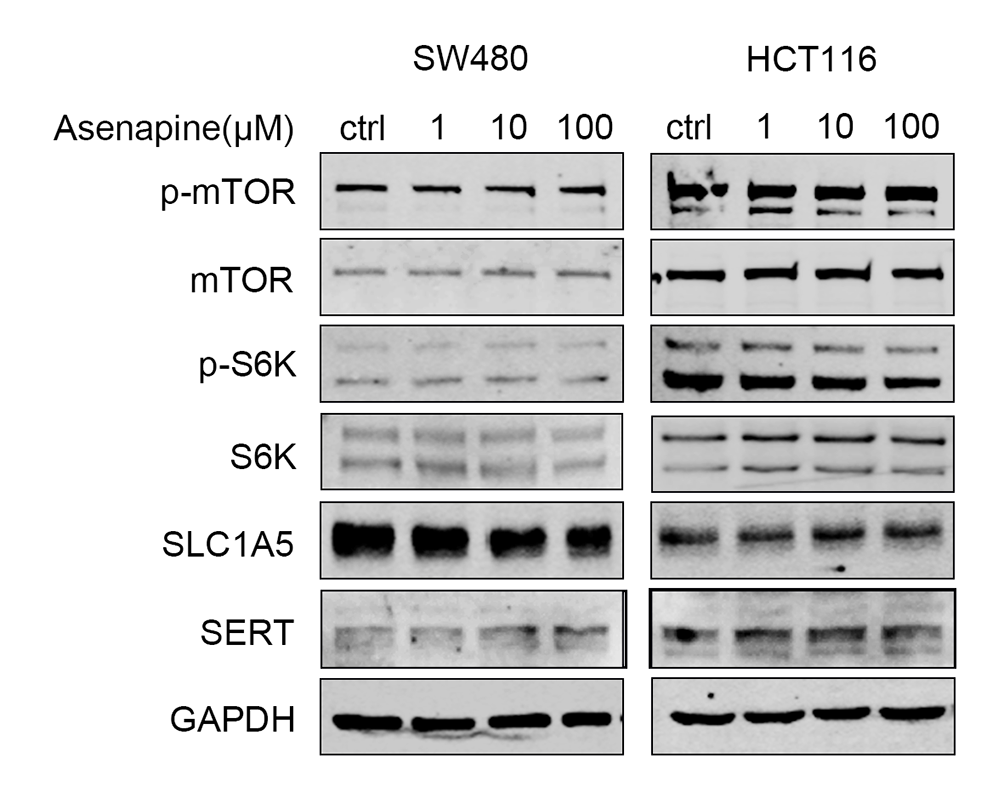

Supplement: Supplementary file 5 — Additional file 5: Figure S5. Serotonin receptor broad-spectrum inhibitors cannot inhibit serotonin activation of Mtor. A WB for mTOR, S6K, SERT and SLC1A5 using SW480 and HCT116 cells treated with increasing concentrations of Asenapine (1-100 μM). [file 13046_2021_1971_MOESM5_ESM.tif]

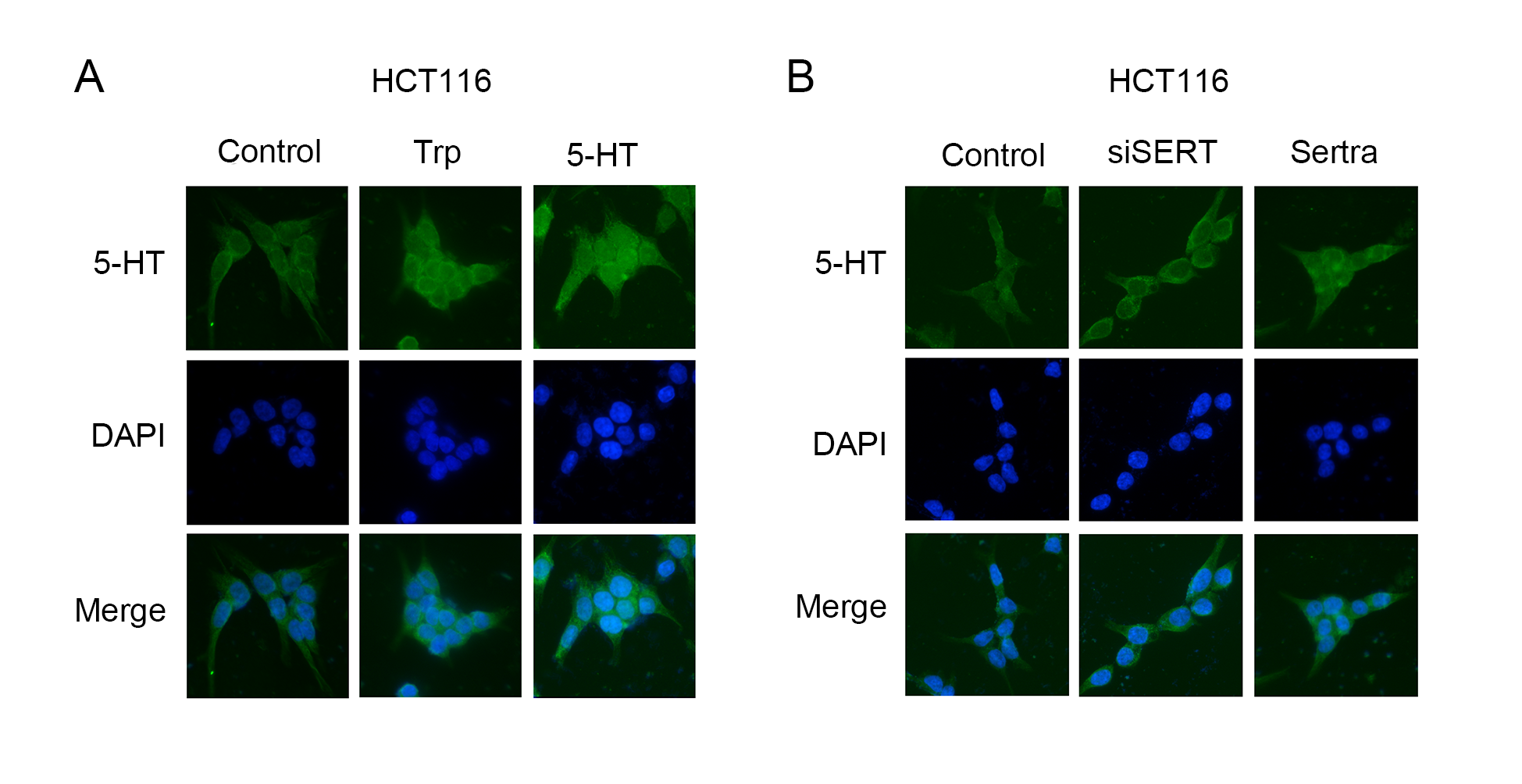

Supplement: Supplementary file 6 — Additional file 6: Figure S6. Endogenous and exogenous serotonin have different subcellular locations. A HCT116 cells were starved with Trp-free and serum-free medium for 24 h after which they were supplied with Trp (75 μM, 9 h) or serotonin (50 μM, 4 h) for 9 h, respectively. Serotonin was demonstrated by anti-serotonin staining (green). DNA was stained with DAPI (blue). Scale bar: 25 μm. B HCT116 cells were transfected with siSERT (48 h) or negative control (48 h) or sertraline (15 μM, 12 h). Serotonin localization was visualized by IF staining with anti-serotonin (green). DNA was stained with DAPI (blue). Scale bar: 25 μm. [file 13046_2021_1971_MOESM6_ESM.tif]

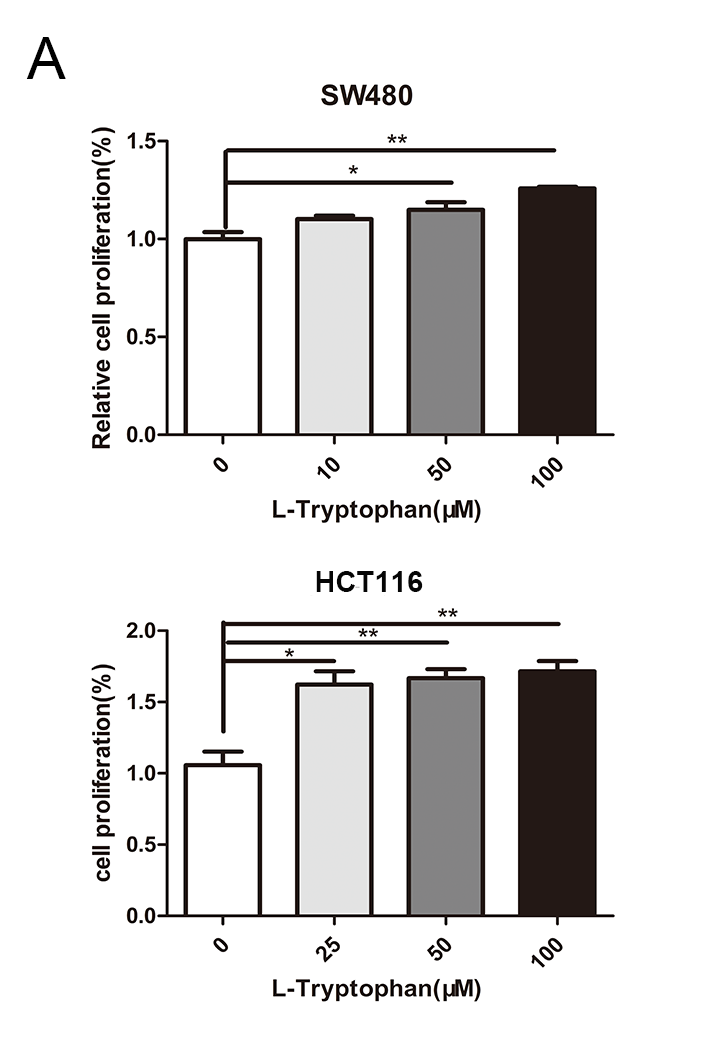

Supplement: Supplementary file 7 — Additional file 7: Figure S7. The effect of tryptophan on the proliferation of colon cancer cells. A SW480 and HCT116 cells were cultured in 96-well plates in DMEM containing 10% FBS and incubated overnight. The next day, cells were starved with Trp-depletion and serum-free medium for 24 h. Thereafter, cells were cultured in a medium supplemented with Trp (10-100 μM) or without Trp for an additional 2 days. Then, their viability was detected using a CCK8 kit. [file 13046_2021_1971_MOESM7_ESM.tif]

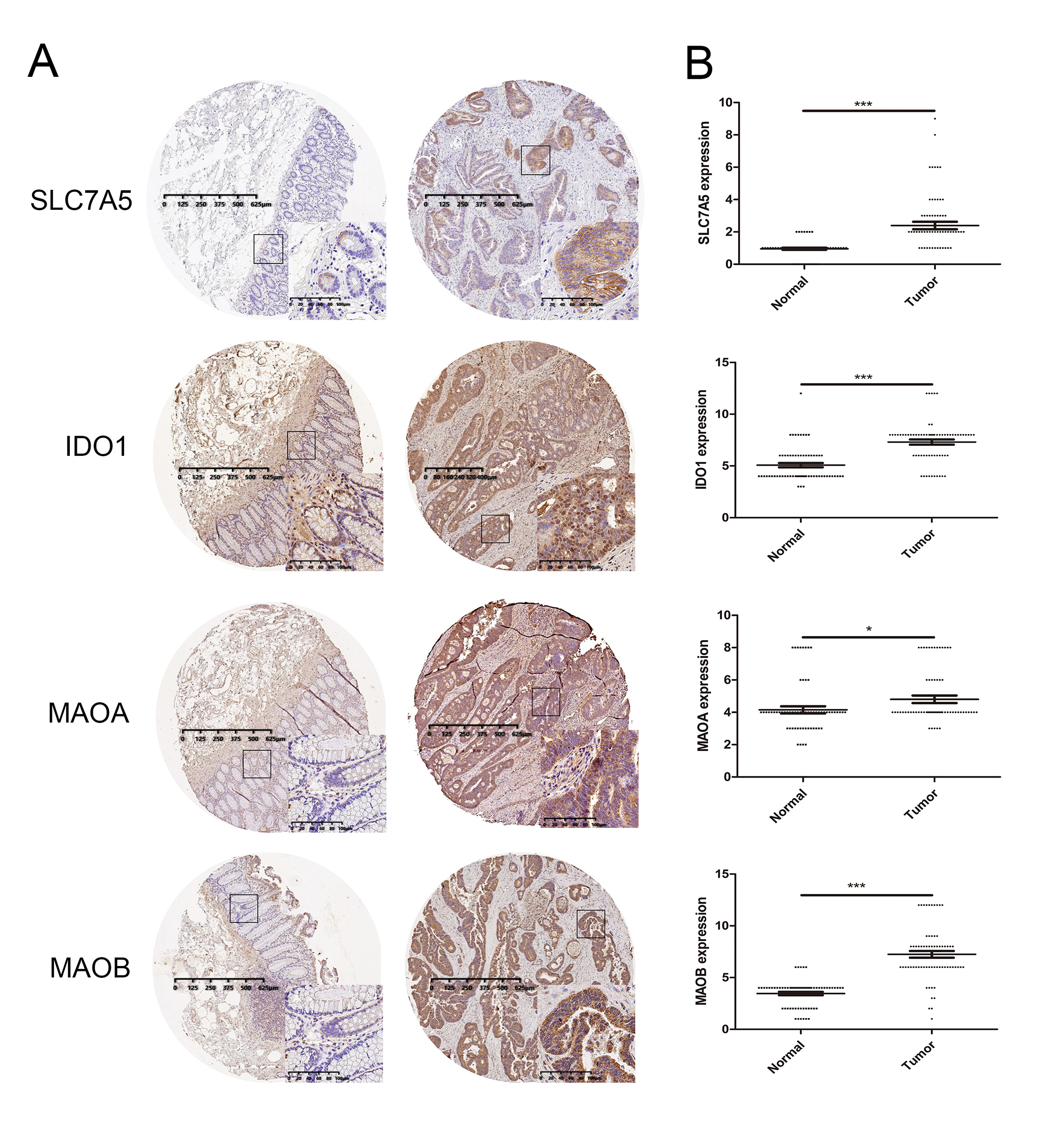

Supplement: Supplementary file 8 — Additional file 8: Figure S8. Expression of SLC7A5, IDO1, MAOA and MAOB in colon cancer tissues. A Representative images of Trp metabolism-related proteins in clinical samples of colon cancer and normal colon tissues. B: IHC analysis of SLC7A5, IDO1, MAOA and MAOB expression in clinical samples of colon cancer tissues and normal colon tissues. The method for assigning IHC scores for each sample was described in the “Materials and methods” section. *** p < 0.001. [file 13046_2021_1971_MOESM8_ESM.tif]

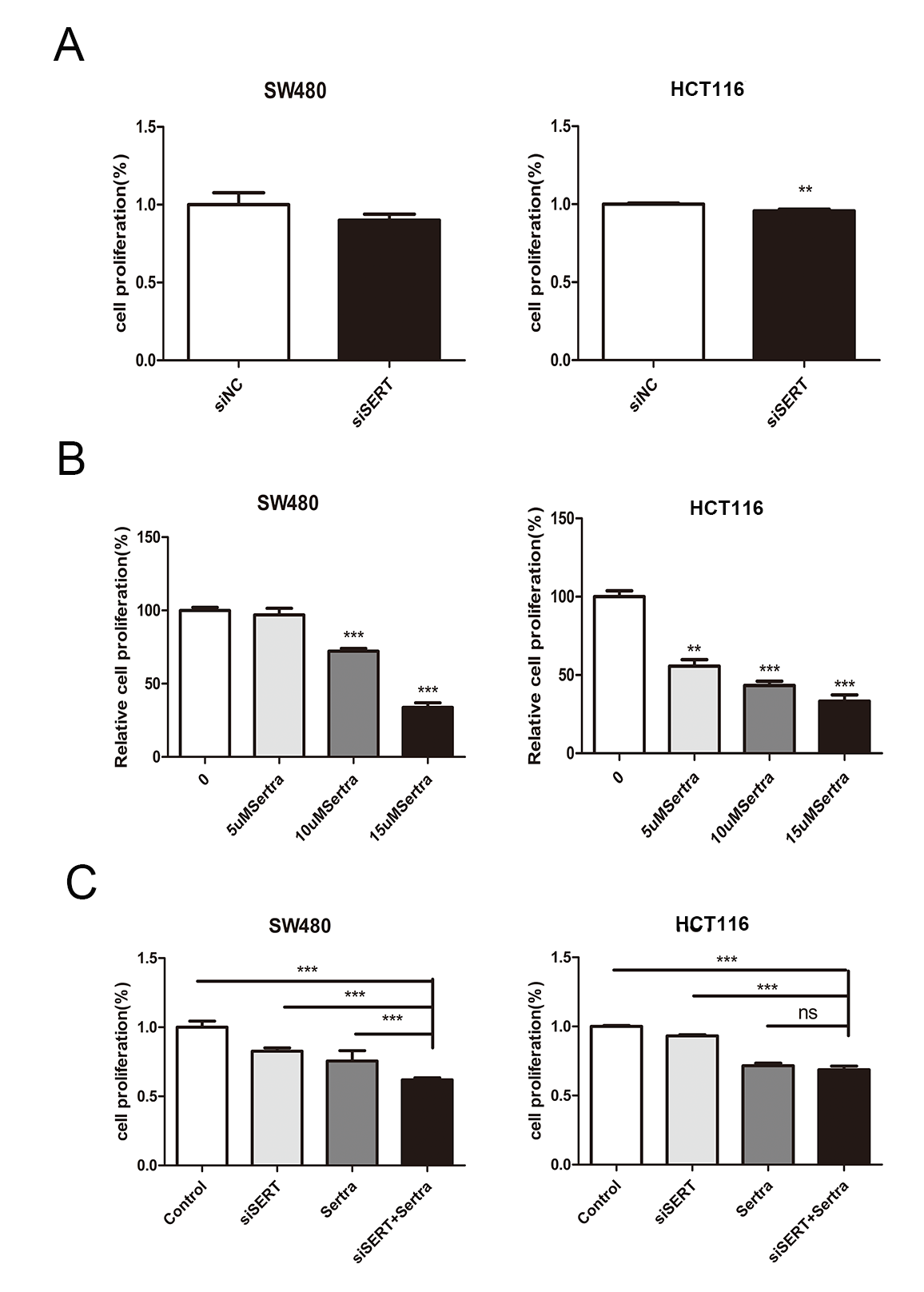

Supplement: Supplementary file 9 — Additional file 9: Figure S9. The effect of silencing and inhibiting SERT on the proliferation of colon cancer cells. A Proliferation assays for SW480 and HCT116 cells transfected with siSERT were performed using the Cell Counting Kit-8 at day 3. B Proliferation assays for SW480 and HCT116 cells treated with increasing concentration of sertraline (5-15 μM) at day 2. C SW480 and HCT116 cells were transfected with siSERT for 24 h and treated with sertraline (10 μM) for another 48 h. The Cell Counting Kit-8 experiment was used to detect cell vitality. Data presented are presented as the mean ± SD, *p < 0.05, **p < 0.01, ***p < 0.001 using the Student’s t test (two-tailed). [file 13046_2021_1971_MOESM9_ESM.tif]

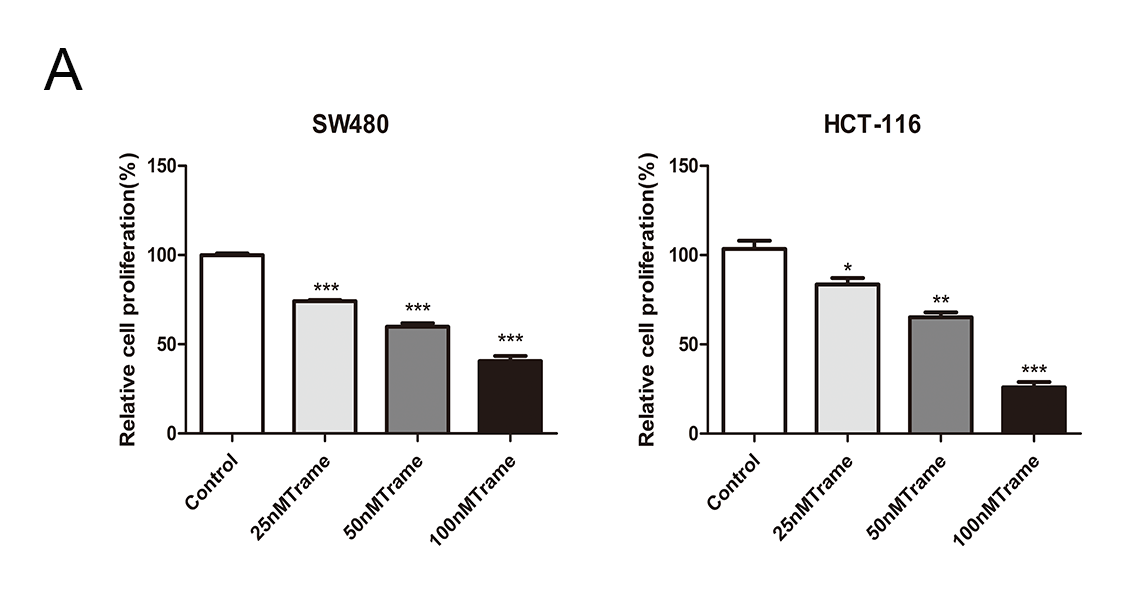

Supplement: Supplementary file 10 — Additional file 10: Figure S10. Effect of trametinib on the proliferation of colon cancer cells. A Proliferation assays were performed on SW480 and HCT116 cells treated with increasing concentrations of trametinib (25-100 nM) at day 2. Data are presented as the mean ± SD, *p < 0.05, **p < 0.01, ***p < 0.001 using Student’s t test (two-tailed). [file 13046_2021_1971_MOESM10_ESM.tif]

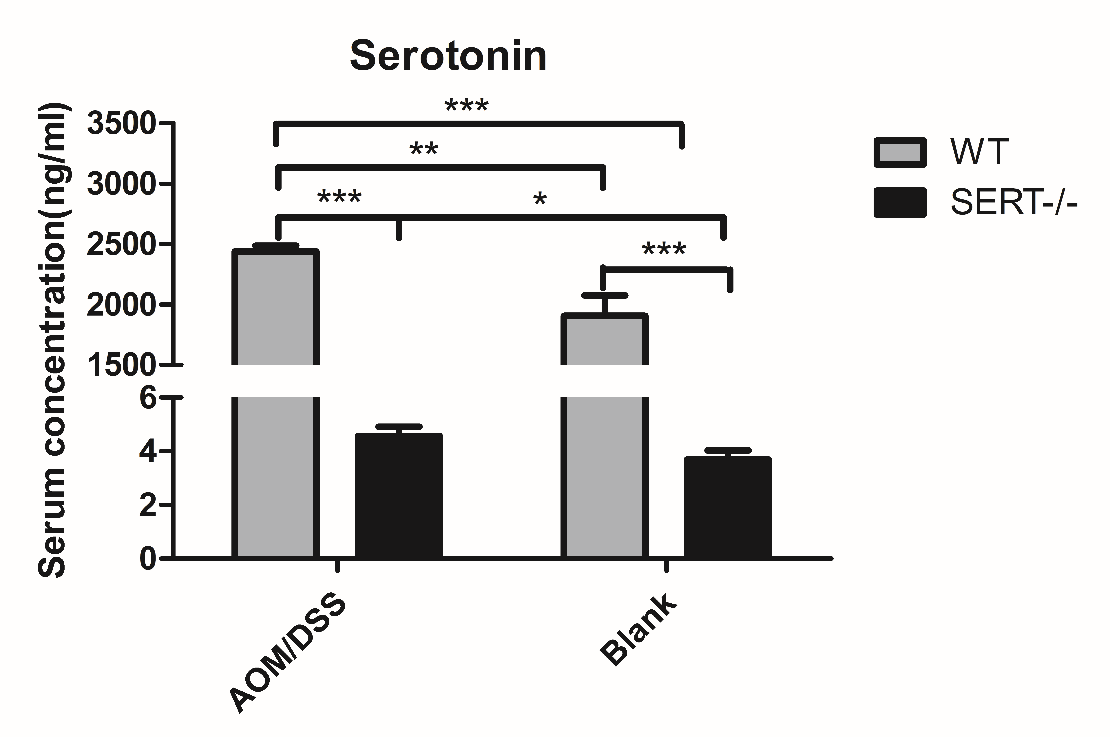

Supplement: Supplementary file 11 — Additional file 11: Figure S11. The effect of knocking-out SERT on plasma serotonin levels in normal mice and in colon cancer mouse model. Serum levels of serotonin in SERT-WT and SERT-KO mice in normal and AOM/DSS group were detected using LCMS/MS. [file 13046_2021_1971_MOESM11_ESM.tif]
