# Supplementary material for: Targeting SERT promotes tryptophan metabolism: mechanisms and implications in colon cancer treatment
Source: J Exp Clin Cancer Res. 2021 May 18;40:173. doi: 10.1186/s13046-021-01971-1 (PMC8132442; doi:10.1186/s13046-021-01971-1)
Supplement: Supplementary file 12 — Additional file 12: Table S1. Primer sequence. [file 13046_2021_1971_MOESM12_ESM.docx]

**Table 1.** Primer sequence

| SERT | Forward | GACAGCCACCTTCCCTTATATC |
| --- | --- | --- |
|  | Reverse | ACCTCAGACACATCTTCATTCC |
| SLC1A5 | Forward | GAGCTGCTTATCCGCTTCTTC |
|  | Reverse | GGGGCGTACCACATGATCC |
| SLC7A5 | Forward | CCGTGAACTGCTACAGCGT |
|  | Reverse | CTTCCCGATCTGGACGAAGC |
| IDO1 | Forward | GAAACTGGAACTGCCTCCTATT |
|  | Reverse | GTCTTCCCAGAACCCTTCATAC |
| TDO2 | Forward | CTCTGGGAGTTGGATTCTGTTC |
|  | Reverse | CAGTTGATCGCAGGTAGTGATAG |
| AFMID | Forward | AGCGGTATCCAAGCAACAAGG |
|  | Reverse | AGACCCCACTCACCAGGAAAA |
| TPH1 | Forward | CGTTCCTCTCTTGGCTGAAC |
|  | Reverse | CTGATGGAAGAAAGCAAGCC |
| GAPDH | Forward | TCGTGGAAGGACTCATGACCA |
|  | Reverse | AGGCAGGGATGATGTTCTGGA |
